# Gold(I)-catalyzed domino cyclization for the synthesis of polyaromatic heterocycles

**DOI:** 10.3762/bjoc.9.297

**Published:** 2013-11-22

**Authors:** Mathieu Morin, Patrick Levesque, Louis Barriault

**Affiliations:** 1 Center for Catalysis Research and Innovation, Department of Chemistry, University of Ottawa, Ottawa, Canada K1T 1B5

**Keywords:** catalysis, cyclization, gold(I), gold catalysis, heterocycles, regioselectivity

## Abstract

Gold(I) complexes have emerged as powerful and useful catalysts for the formation of new C–C, C–O and C–N bonds. Taking advantage of the specificity of [IPrAuNCMe][SbF_6_] complexes to favor the 5-*exo*-*dig* cyclization over the 6-*endo*-*dig* pathway, we report a high yielding and efficient method to generate substituted polyaromatic heterocycles under remarkably mild reaction conditions.

## Introduction

**Scheme 1:**
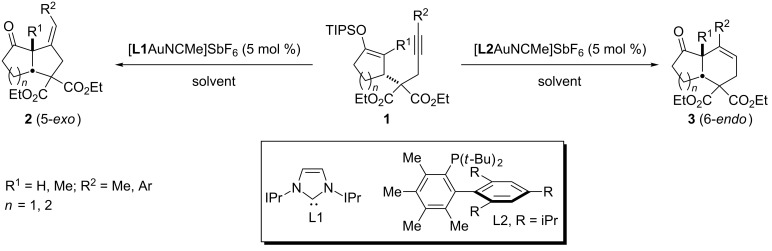
Gold(I)-catalyzed carbocyclization.

In the last decade, phosphino and NHC–gold complexes have become prominent catalysts for the addition of nucleophiles to alkenes, alkynes and allenes [1–11]. Owing to the high affinity of gold(I) complexes to C–C π-systems in the presence of other functional groups combined by its predictable reactivity pattern, the gold(I)-catalyzed reaction provides tremendous opportunities for the discovery of new and useful reactions [12]. Recently, we [13] and other groups [14–17] reported that divergent pathway can be obtained by modulating the steric and electronic properties of the gold(I) catalyst (Scheme 1). The ancillary ligand plays a direct role in the regioselectivity for the first bond formation rather than via a common intermediate created after an initial bond formation [18]. Indeed, the cyclization of cyclic enol ether **1** using σ-donor ligands such as IPr (**L1**) [19] was exceptionally selective for the *5-exo*-*dig* pathway (**1**→**2**) whereas bulky Me_4_XPhos (**L2**) [12] gave mainly *6-endo*-*dig*-cyclized product **3**.

## Results and Discussion

**Scheme 2:**
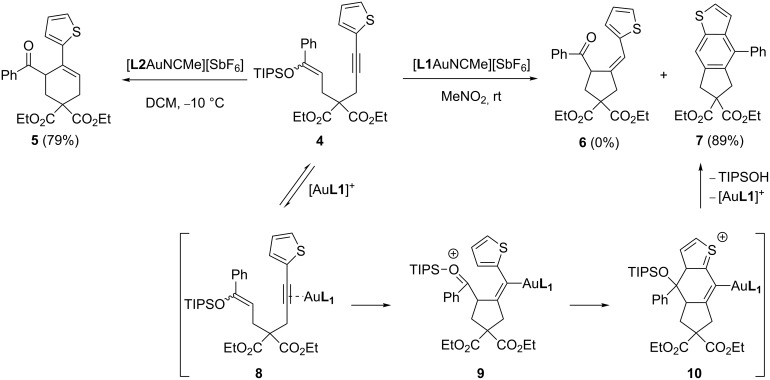
Proposed mechanism for the gold(I)-catalyzed cyclization.

During the course of our investigation, we examined the cyclization of non-cyclic enol ethers. As expected, the cyclization of enol ether **4** using [**L2**AuNCMe][SbF_6_] in dichloromethane afforded the cyclohexene **5** in 79% yield (Scheme 2). However, the anticipated *5-exo*-*dig* product **6** was not observed when the catalyst [**L1**AuNCMe][SbF_6_] was utilized. Instead, the benzothiophene **7** was isolated in 89% yield. The formation of this unforeseen product can be explained by the proposed mechanism illustrated in Scheme 2. The gold(I) complexation of alkyne **4** triggers the *5-exo*-*dig* cyclization to produce intermediate **9**. At this point, a nucleophilic addition of the thiophene unit to the carboxonium provides the sulfonium **10** which upon protodeauration and aromatization gives **7** [20–25]. Other polar solvents such as acetone and dichloromethane were employed without much success. One might consider that the high polarity of nitromethane helps to alleviate the charge build-up at the cationic cyclization transition state.

**Scheme 3:**
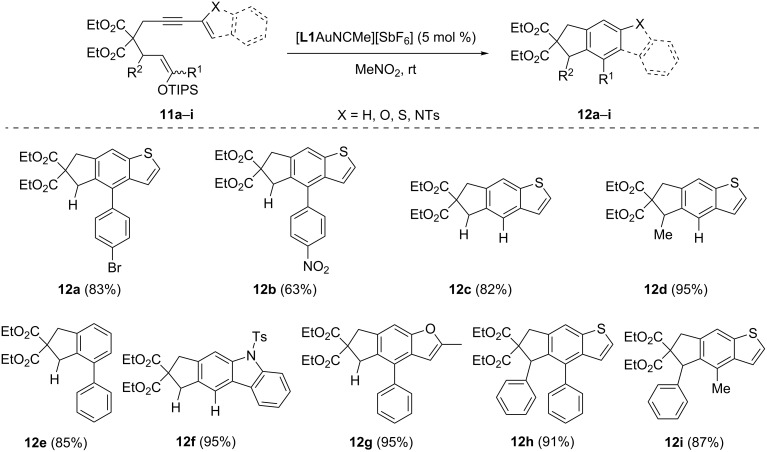
Gold-catalyzed 5-*exo*-*dig* carbocyclization cascade.

Substituted aromatic compounds have a fundamental importance in organic and medicinal chemistry as well as in materials. Although there are many methods to functionalize aromatic rings, one can recognize that the above transformation represents an attractive and complementary approach for the synthesis of substituted aromatic rings. Taking advantage of the high regiospecificity of [**L1**AuNCMe][SbF_6_] associated with alkyne activation, we examined the scope of the reaction using various substituted alkynes (Scheme 3). Gold(I)-catalyzed cyclizations of the enol ether **11a** (R^1^ = *p*-BrC_6_H_4_, R^2^ = H) gave the corresponding benzothiophene **12a** in 83% yield. The use of electron-poor silyl enol ether **11b** (R^1^ = *p*-NO_2_C_6_H_4_, R^2^ = H) gave the desired product **12b**, albeit in lower yield of 63%. Di- and trisubstituted silyl enol ethers **11c** (R^1^ and R^2^ = H) and **11d** (R^1^ = H and R^2^ = Me) were converted to benzothiophenes **12c** and **12d** in 82% and 95% yield, respectively. The synthesis of substituted hydrindene **12e** was also achieved in 85% yield from monosubstituted enyne **11e** (R^1^ = Ph, R^2^ = H). Substituted enynes bearing heterocycles such as indole **11f** (R^1^ and R^2^ = H) and furan **11g** (R^1^ = Ph and R^2^ = H) were effectively transformed to the desired carbazole **12f** and benzofuran **12g** in 95% yields. It can be noticed that large substituents at R^1^ and R^2^ did not affect the efficiency of the reaction. The gold(I)-catalyzed cyclization of **11h** (R^1^ = R^2^ = Ph) and **11i** (R^1^ = Ph and R^2^ = Me) provided the corresponding benzothiophenes **12h** and **12i** in 91% and 87% yield, respectively.

## Conclusion

**Figure 1:**
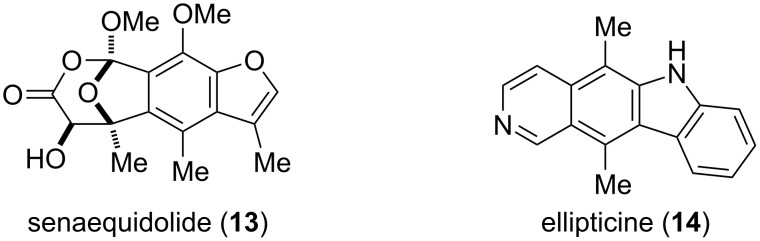
Structure of senaequidolide (**13**) and ellipticine (**14**).

In summary, we have developed a mild and efficient gold(I)-catalyzed 5-*exo*-*dig* polycyclization cascade to prepare an array of substituted aromatic compounds such as benzofuran, benzothiophene, carbazole and hydrindene in high yields. The use of σ-donor ligands such as IPr (**L1**) was exceptionally selective for the *5-exo*-*dig* pathway. This Au(I)-catalyzed cyclization occurring in cascade provides a direct access to synthetically useful motifs commonly found in natural products and important medicinally compounds. The application of this method in the total syntheses of senaequidolide (**13**) [26] and ellipticine (**14**) [27–28] are currently underway and will be reported in due course (Figure 1).

## Experimental

**General experimental procedure for the Au(I)-catalyzed cyclization**: In a flask equipped with a magnetic stirrer was added the silyl enol ether **11** (0.101 mmol) followed by nitromethane (1 mL) and [**L1**AuNCMe][SbF_6_] (0.005 mmol). After stirring overnight, the reaction mixture was concentrated in vacuo and the crude mixture was purified by flash chromatography (1–5% ethyl acetate/hexanes) to give the desired cyclized product **12**.

## Supporting Information

File 1 Materials and methods, experimental procedures for **4** and **11a**–**i**, characterization data for **5**, **7** and **12a**–**i**, ^1^H and ^13^C NMR spectra for all cyclized compounds.
