# Supplementary material for: Gold(I)-catalyzed domino cyclization for the synthesis of polyaromatic heterocycles
Source: Beilstein J Org Chem. 2013 Nov 22;9:2625–8. doi: 10.3762/bjoc.9.297 (PMC3869369; doi:10.3762/bjoc.9.297)
Supplement: File 1 — Materials and methods, experimental procedures for 4 and 11a–i, characterization data for 5, 7 and 12a–i, 1H and 13C NMR spectra for all cyclized compounds. [file Beilstein_J_Org_Chem-09-2625-s001.pdf]

Supporting Information

for

Gold(I)-catalyzed domino cyclization for the synthesis of polyaromatic heterocycles

Mathieu Morin, Patrick Levesque and Louis Barriault*

Address: Center for Catalysis Research and Innovation, Department of Chemistry, University of Ottawa, Ottawa, Canada K1T 1B5

Email: Louis Barriault - lbarriau@uottawa.ca

* Corresponding author

Materials and methods, experimental procedures for **4** and **11a–i**, characterization data for **5**, **7** and **12a–i**, ^{1}H and ^{13}C NMR spectra for all cyclized compounds

General Information

All reactions were performed under argon atmosphere in flame-dried glassware equipped with a magnetic stir bar and a rubber septum, unless otherwise indicated. Most solvents were filtered using LC Technology Solutions INC. Solvent Systems. All other commercial reagents were used without further purification, unless otherwise noted. Reactions were monitored by thin-layer chromatography (TLC) analysis of aliquots using glass sheets precoated (0.2 mm layer thickness) with silica gel 60 F254 (E. Merck). Thin-layer chromatography plates were viewed under UV light and stained with potassium permanganate or *p*-anisaldehyde staining solution. Column chromatography was carried out with silica gel 60 (230–400 mesh, Merck). Yields refer to products isolated after this purification, unless otherwise stated. Proton nuclear magnetic resonance (^{1}H NMR) spectra were recorded on Bruker AMX 300 MHz and Bruker AMX 400 MHz. NMR samples were dissolved in $CDCl_3$ (unless specified otherwise) and chemical shifts are reported in ppm referenced to residual undeuterated solvent. Data are reported as follows: chemical shift, multiplicity, coupling, integration, where multiplicity (s = singlet, d = doublet, dd = doublet of doublets, ddd = doublet of doublets of doublets, dt = doublet of triplets, ddt = doublet of doublets of triplets, dq = doublet of quartets, br = broad signal, t = triplet, td = triplet of doublets, tt = triplet of triplets, tquin = triplet of quintets, q = quartet, qd = quartet of doublets, quin = quintet, m = multiplet, or otherwise noted), coupling constant. Carbon nuclear magnetic resonance (^{13}C NMR) spectra were recorded same Bruker instruments as in ^{1}H NMR using 75 MHz or 100 MHz. IR spectra were recorded with a Bomem Michaelson 100 FTIR

spectrometer. HRMS were obtained on a Kratos Analytical Concept instrument (University of Ottawa Mass Spectrum Centre).

General procedure for the formation of 15a–d

To a flame-dried round-bottomed flask equipped with a magnetic stirrer under argon was added $Pd(PPh_3)_4$ (0.063 mmol, 0.05 equiv) and CuI (0.126 mmol, 0.10 equiv). Benzene (12.6 mL, 0.1 M) was added and the mixture was degassed (sparged) for 15 min. During the degassing, the aryl (or vinyl) halide (1.89 mmol, 1.5 equiv) was added followed by diethyl 2-(prop-2-yn-1-yl)malonate (1.26 mmol, 1 equiv). Once the degassing is finished, diethylamine (6.31 mmol, 5 equiv) is added and the solution is stirred at room temperature overnight. After workup and evaporation of the solvent, the residue was purified by column chromatography to give the desired product **15**.

General procedure for the formation of 4, 11a–i

Solution 1: To a flame-dried round-bottomed flask equipped with a magnetic stirrer under argon was added NaH (7.13 mmol, 2 equiv) in THF (17 mL) followed by the addition of compound **15** (5.35 mmol, 1.5 equiv) drop wise and the reaction was stirred for 30 min at room temperature.

Solution 2: In a separate round-bottomed flask equipped with magnetic stirrer under argon was added the enone (3.57 mmol, 1 equiv) followed by THF (17 mL). The solution was cooled to −78 °C and TIPSOTf (5.35 mmol, 1.5 equiv) was added slowly. The reaction mixture was stirred for 5 min, then Me_2S (10.70 mmol, 3 equiv) was added to the solution drop wise along the side of the flask, and the solution is stirred for 25 min

at −78 °C. Solution 1 was added via cannula to solution 2 in a drop wise manner. The mixture was then stirred for 1 h as the bath warms to room temperature. The reaction mixture was quenched with a saturated aqueous $NaHCO_3$ and extracted with EtOAc. The fractions were concentrated and the residue was purified by column chromatography (EtOAc/hexanes) to give the desired product.

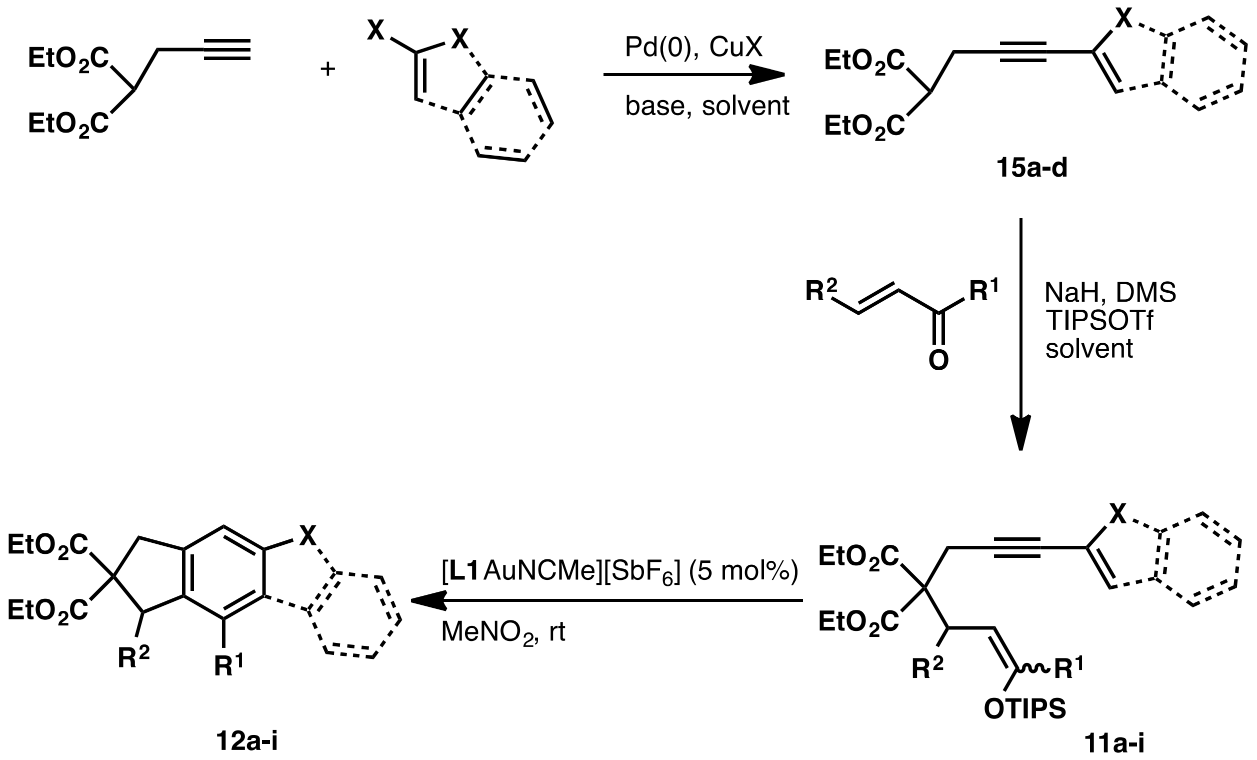

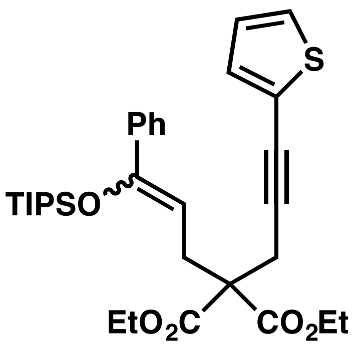

(4): ^{1}H NMR (400 MHz, $CDCl_3$) δ 7.40 (dd, *J* = 7.84, 1.67 Hz, 2 H) 7.17 - 7.35 (m, 3 H) 7.15 (dd, *J* = 5.19, 1.08 Hz, 1 H) 7.06 (dd, *J* = 3.53, 0.98 Hz, 1 H) 6.90 (dd, *J* = 5.14, 3.67 Hz, 1 H) 4.84 (t, *J* = 6.96 Hz, 1 H) 4.04 - 4.29 (m, 4 H) 3.06 (s, 2 H) 3.04 (d, *J* = 6.96 Hz, 2 H) 1.24 (t, *J* = 7.15 Hz, 6 H) 0.98 - 1.05 (m, 21 H) ppm; ^{13}C NMR (101 MHz , $CDCl_3$) δ 170.2 (2 X C), 153.4 (C), 139.9 (C), 131.5 (CH), 127.9 (2 X CH), 127.8 (CH), 126.7 (CH), 126.3 (CH), 126.2 (2 X CH), 123.5 (C), 103.7 (CH), 89.1 (C), 76.3 (C), 61.6 (2 X CH_2), 57.3 (C), 29.7 (CH_2), 24.5 (CH_2), 17.9 (6 X CH_3), 14.1 (2 X CH_3), 12.3 (3 X CH) ppm. IR (neat, cm^{-1}): 2942, 2865, 1733; HRMS (EI) *m*/*z* calcd for $C_{32}H_{44}O_5SSi$ $[M]^+$: 568.2679, found: 568.2657.

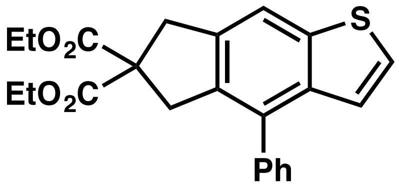

(7): ^{1}H NMR (400 MHz, $CDCl_3$): δ 7.66 (s, 1 H) 7.33 - 7.52 (m, 5 H) 7.27 (d, *J* = 5.58 Hz, 1 H) 7.09 (dd, *J* = 5.58, 0.69 Hz, 1 H) 4.16 (qd, *J* = 7.09, 0.59 Hz, 3 H) 3.72 (s, 2 H) 3.51 (s, 2 H) 1.20 (t, *J* = 7.15 Hz, 6 H) ppm; ^{13}C NMR (100 MHz, $CDCl_3$): δ 171.5 (2 X C), 139.5 (C), 139.0 (C), 138.1 (C), 137.5 (C), 135.2 (C), 133.3 (C), 129.3(2 X CH), 128.4 (2 X CH), 127.4 (CH), 125.3 (CH), 123.1 (CH), 117.0 (CH), 61.7 (2 X CH_2), 61.0 (C), 40.4 (CH_2), 39.3 (CH_2), 14.0 (2 X CH_3) ppm; IR (neat, cm^{-1}): 2360, 1733; HRMS (EI) *m*/*z* calcd for $C_{23}H_{22}O_4S$ $[M]^+$: 394.1239, found: 394.1246.

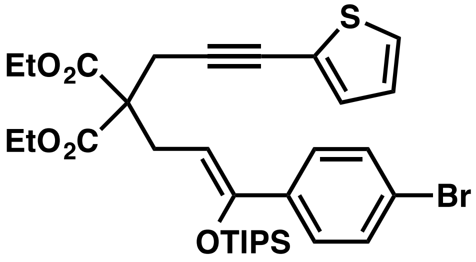

(11a): Characterized as *E*/*Z* mixture. **Major Isomer** ^{1}H NMR (400 MHz, $CDCl_3$) δ 7.46 - 7.37 (m, 2 H), 7.34 - 7.23 (m, 2 H), 7.17 (dd, *J* = 1.2, 5.2 Hz, 1 H), 7.06 (dd, *J* = 1.1, 3.5 Hz, 1 H), 6.91 (dd, *J* = 3.5,

5.2 Hz, 1 H), 4.89 (t, J = 7.0 Hz, 1 H), 4.30 - 4.08 (m, 4 H), 3.07 (s, 2 H), 3.04 (d, J = 7.0 Hz, 2 H), 1.25 (t, J = 7.1 Hz, 6 H), 1.14 - 0.98 (m, 21 H) ppm; ^{13}C NMR (101 MHz , $CDCl_3$) δ 170.1 (2 X C), 152.4 (C), 138.9 (C), 131.6 (CH), 131.2 (2 X CH), 130.2 (CH), 127.8 (2 X CH), 126.8 (CH), 126.4 (CH), 123.5 (C), 121.8(C), 104.6 (CH), 89.0 (C), 76.5 (C), 61.7 (CH_2), 57.3 (C), 29.9 (CH_2), 24.6 (CH_2), 18.0 (6 X CH_3), 13.5 (2 X CH_3), 12.6 (3 X CH) ppm. **Minor Isomer** ^{1}H NMR (400 MHz, $CDCl_3$) δ 7.46 - 7.37 (m, 2 H), 7.34 - 7.23 (m, 2 H), 7.17 (dd, J = 1.2, 5.2 Hz, 1 H), 7.06 (dd, J = 1.1, 3.5 Hz, 1 H), 6.91 (dd, J = 3.5, 5.2 Hz, 1 H), 4.79 (t, J = 7.7 Hz, 1 H), 4.30 - 4.08 (m, 4 H), 3.00 (s, 2 H), 2.93 (d, J = 7.7 Hz, 2 H), 1.22 (t, J = 7.1 Hz, 6 H), 1.14 - 0.98 (m, 21 H) ppm; ^{13}C NMR (101 MHz, $CDCl_3$) δ 169.9 (2 X C), 152.5 (C), 136.4 (C), 131.7 (CH), 131.2 (2 X CH), 129.8 (CH), 127.8 (2 X CH), 126.8 (CH), 126.5 (CH), 123.2 (C), 122.1 (C), 102.3 (CH), 88.4 (C), 76.6 (C), 61.7 (CH_2), 57.6 (C), 30.5 (CH_2), 23.8 (CH_2), 18.1 (6 X CH_3), 14.2 (2 X CH_3), 12.3 (3 X CH) ppm; IR (neat, cm^{-1}): 3103, 1732; HRMS (EI) *m*/*z* calcd for $C_{32}H_{43}BrO_5SSi$ $[M]^+$: 646.1784, found: 646.1741.

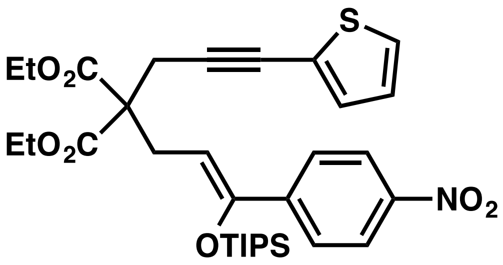

(11b): ^{1}H NMR (400 MHz, $CDCl_3$) δ 8.13 (d, J = 8.9 Hz, 2 H), 7.55 (d, J = 8.9 Hz, 2 H), 7.16 (dd, J = 1.2, 2.3 Hz, 1 H), 7.05 (dd, J = 1.1, 3.6 Hz, 1 H), 6.89 (t, J = 3.7 Hz, 1 H), 5.11 (t, J = 7.0 Hz, 1 H), 4.29 - 4.11 (m, 4 H), 1.27 (t, J = 7.2 Hz, 6 H), 1.04 - 1.00 (m, 21 H) ppm; ^{13}C NMR (101 MHz, $CDCl_3$) δ 169.9 (C), 168.0 (C), 151.4 (C), 147.2 (C), 146.1 (C), 131.6 (CH), 129.2 (CH), 126.8 (CH), 126.4 (2 X CH), 123.5 (2 X CH), 108.0 (CH), 89.5 (C), 88.6 (C), 75.6 (C), 61.8 (2 X CH_2), 57.0 (C), 30.0 (CH_2), 24.7 (CH_2), 17.9 (6 X CH_3), 14.1 (2 X

CH_3), 12.3 (3 X CH) ppm; IR (neat, cm^{-1}): 3105, 1734; HRMS (EI) *m/z* calcd for $C_{32}H_{43}NO_7SSi$ $[M]^+$: 613.2529, found: 613.2537.

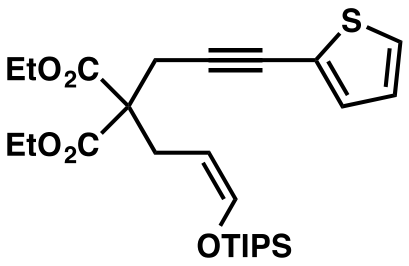

(11c): ^1H NMR (400 MHz, $CDCl_3$) δ 7.17 (dd, *J* = 1.2, 5.2 Hz, 1 H), 7.09 (dd, *J* = 1.2, 3.6 Hz, 1 H), 6.92 (dd, *J* = 3.6, 5.2 Hz, 1 H), 6.45 (d, *J* = 11.7 Hz, 1 H), 4.80 (td, *J* = 8.1, 11.7 Hz, 1 H), 4.29 - 4.13 (m, *J* = 2.8, 7.1, 7.1, 7.1 Hz, 4 H), 3.02 (s, 2 H), 2.69 (dd, *J* = 1.0, 8.0 Hz, 2 H), 1.26 (t, *J* = 7.2 Hz, 6 H), 1.17 - 1.09 (m, 3 H), 1.06 (d, *J* = 6.7 Hz, 18 H) ppm; ^{13}C NMR (101 MHz, $CDCl_3$) δ 170.1 (2 X C), 144.5 (CH), 131.6 (CH), 126.9 (CH), 126.5 (CH), 123.6 (C), 103.2 (CH), 88.9 (C), 76.5 (C), 61.7 (2 X CH_2), 57.5 (C), 30.7 (CH_2), 23.7 (CH_2), 17.8 (6 X CH_3), 14.2 (2 X CH_3), 12.1 (3 X CH) ppm; IR (neat, cm^{-1}): 1734; HRMS (EI) *m/z* calcd for $C_{26}H_{40}O_5SSi$ $[M]^+$: 492.2366, found: 492.2385.

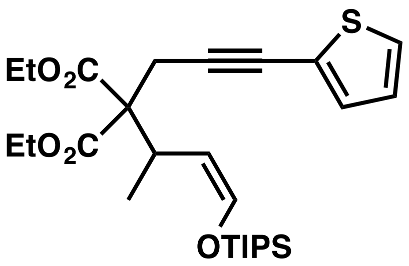

(11d): ^1H NMR (400 MHz, $CDCl_3$) δ 7.15 (dd, *J* = 1.0, 5.1 Hz, 1 H), 7.06 (d, *J* = 3.5 Hz, 1 H), 6.90 (dd, *J* = 3.6, 5.1 Hz, 1 H), 6.47 (d, *J* = 11.7 Hz, 1 H), 4.84 (dd, *J* = 10.4, 11.6 Hz, 1 H), 4.20 (q, *J* = 7.1 Hz, 4 H), 3.00 (q, *J* = 17.1 Hz, 2 H), 3.08 - 2.90 (m, 1 H), 1.25 (t, *J* = 7.4 Hz, 6 H), 1.16 (d, *J* = 6.9 Hz, 3 H), 1.14 - 0.98 (m, 21 H) ppm; ^{13}C NMR (101 MHz, $CDCl_3$) δ 170.0 (C), 169.6 (C), 142.7 (CH), 131.3 (CH), 126.7 (CH), 126.2 (CH), 123.6 (C), 110.6 (CH), 89.6 (C), 76.3 (C), 61.3 (2 X CH_2), 60.8 (C), 36.3 (CH), 25.4 (CH_2), 18.7 (CH_3) 17.7 (6 X CH_3), 14.1 (2 X CH_3) , 12.0 (3 X CH) ppm; IR (neat, cm^{-1}): 2942, 2866, 1728; HRMS (EI) *m/z* calcd for $C_{27}H_{42}O_5SSi$ $[M]^+$: 506.2522, found: 506.2526.

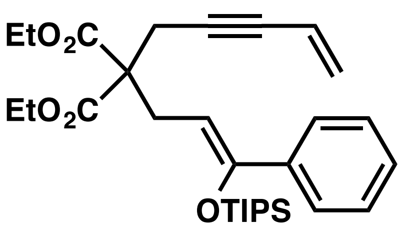

(11e): ^{1}H NMR (400 MHz, $CDCl_3$) δ 7.62 - 7.25 (m, 5 H), 5.75 - 5.64 (m, 1 H), 5.58 - 5.47 (m, 1 H), 5.34 (s, 1 H), 4.79 (t, *J* = 7.0 Hz, 1 H), 4.29 - 4.00 (m, 4 H), 3.07 - 2.83 (m, 4 H), 1.22 (t, *J* = 6.8 Hz, 6 H), 1.13 - 0.97 (m, 21 H) ppm; ^{13}C NMR (101 MHz, $CDCl_3$) δ 170.2 (2 X C), 153.3 (C), 139.9 (C), 127.9 (2 X CH), 127.8 (CH), 126.3 (CH_2) 126.2 (2 X CH), 117.2 (CH), 103.7 (CH), 85.6 (C), 81.9 (C), 61.5 (2 X CH_2), 57.2 (C), 29.5 (CH_2), 24.0 (CH_2), 17.9 (6 X CH_3), 14.1 (2 X CH_3), 13.5 (3 X CH) ppm; IR (neat, cm^{-1}): 2943, 1733; HRMS (EI) *m*/*z* calcd for $C_{30}H_{44}O_5Si$ $[M]^+$: 512.2958, found: 512.2919.

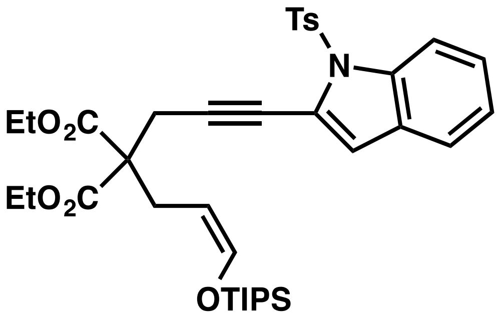

(11f): ^{1}H NMR (400 MHz, $CDCl_3$) δ 8.15 (d, *J* = 8.5 Hz, 1 H), 7.82 (d, *J* = 8.3 Hz, 2 H), 7.39 (d, *J* = 7.7 Hz, 1 H), 7.35 - 7.28 (m, 1 H), 7.23 - 7.15 (m, 3 H), 6.75 (s, 1 H), 6.55 (d, *J* = 11.7 Hz, 1 H), 4.82 (d, *J* = 11.7 Hz, 1 H), 4.22 (dq, *J* = 2.7, 7.1 Hz, 4 H), 3.16 (s, 2 H), 2.80 (d, *J* = 7.9 Hz, 2 H), 2.32 (s, 3 H), 1.25 (t, *J* = 7.1 Hz, 6 H), 1.17 - 0.98 (m, 21 H) ppm; ^{13}C NMR (101 MHz, $CDCl_3$) δ 170.0 (2 X C), 144.8 (C), 144.7 (CH), 136.0 (C), 135.7 (C), 129.7 (2 X CH), 128.9 (C), 127.1 (2 X CH), 125.7 (CH), 123.7 (CH), 120.8 (CH), 117.2 (CH), 114.7 (CH), 103.1 (CH), 93.0 (C), 74.2 (C), 61.7 (2 X CH_2), 57.5 (C), 30.6 (CH_2), 23.8 (CH_2), 21.6 (C), 17.7 (6 X CH_3), 14.1 (2 X CH_3), 12.3 (CH_3), 11.9 (3 X CH) ppm; IR (neat, cm^{-1}): 3109, 1731; HRMS (EI) *m*/*z* calcd for $C_{37}H_{49}NO_7SSi$ $[M]^+$: 679.2999, found: 679.2974.

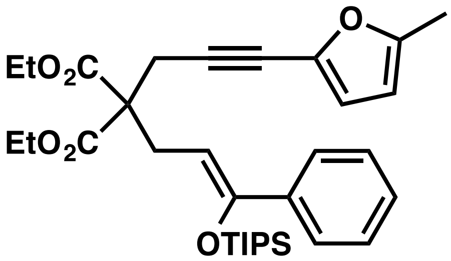

(11g): Characterized as *E*/*Z* mixture. **Major Isomer** ^{1}H NMR (400 MHz, $CDCl_3$) δ 7.46 - 7.33 (m, 2 H), 7.31 - 7.16 (m, 3 H), 6.31 (d, *J* = 3.1 Hz, 1 H), 5.89 (d, *J* = 0.9, 3.2 Hz, 1 H), 4.82 (t, *J* = 7.0 Hz, 1 H), 4.30 - 4.00 (m, 4 H), 3.06 (s, 2 H), 3.03 (d, *J* = 7.1 Hz, 2 H), 2.24 (s, 3 H), 1.23 (t, *J* = 7.1 Hz, 6 H), 1.14 - 0.95 (m, 21 H) ppm; ^{13}C NMR (101 MHz, $CDCl_3$) δ 170.1 (2 X C), 153.4 (C), 152.9 (C), 139.9 (C), 135.4 (C), 127.9 (2 X CH), 127.8 (CH), 126.2 (2 X CH), 115.6 (CH), 106.6 (CH), 103.7 (CH), 88.8 (C), 73.9 (C), 61.6 (2 X CH_2), 57.2 (C), 29.6 (CH_2), 24.3 (CH_2), 17.9 (6 X CH_3), 14.0 (2 X CH_3), 13.4 (CH_3), 12.3 (3 X CH) ppm. **Minor Isomer** ^{1}H NMR (400 MHz, $CDCl_3$) δ 7.46 - 7.33 (m, 2 H), 7.31 - 7.16 (m, 3 H), 6.18 (d, *J* = 3.1 Hz, 1H), 5.86 (dd, *J* = 0.9, 3.2 Hz, 1 H), 4.77 (t, *J* = 7.7 Hz, 1 H), 4.30 - 4.00 (m, 4 H), 3.01 (s, 2 H), 2.92 (d, *J* = 7.7 Hz, 2 H), 2.24 (s, 3 H), 1.17 (t, *J* = 7.1 Hz, 6 H), 1.14 - 0.95 (m, 21 H) ppm; ^{13}C NMR (101 MHz, $CDCl_3$) δ 169.9 (2 X C), 153.4 (C), 152.8 (C), 137.4 (C), 135.2 (C), 128.5 (2 X CH), 128.1 (CH), 127.8 (2 X CH), 115.6 (CH), 106.6 (CH), 101.6 (CH), 88.3 (C), 74.0 (C), 61.8 (2 X CH_2), 57.4 (C), 30.4 (CH_2), 23.7 (CH_2), 17.7 (6 X CH_3), 14.0 (2 X CH_3), 13.8 (CH_3), 12.6 (3 X CH) ppm; IR (neat, cm^{-1}): 3106, 1734; HRMS (EI) *m*/*z* calcd for $C_{33}H_{46}O_6Si$ $[M]^+$: 566.3064, found: 566.3080.

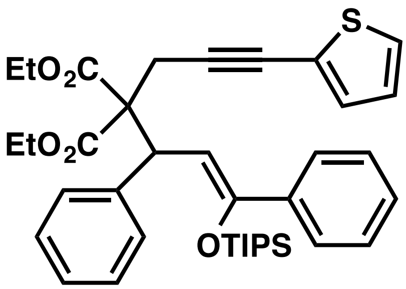

(11h) Characterized as *E*/*Z* mixture. **Major Isomer:** ^{1}H NMR (400 MHz, $CDCl_3$) δ 7.48 - 7.38 (m, 1 H), 7.35 (s, 1 H), 7.34 - 7.16 (m, 8 H), 7.13 (t, *J* = 3.2 Hz, 1 H), 6.88 (q, *J* = 3.0 Hz, 2 H), 5.88 (d, *J* = 11.1 Hz, 1 H), 4.39 (d, *J* = 11.1 Hz, 1 H), 4.28 - 4.04 (m, 4 H), 2.95 (d, *J* = 17.0 Hz, 1 H), 2.80 (d, *J* = 17.0 Hz, 1 H), 1.22 (t, *J* = 7.2 Hz, 6 H), 1.10 - 0.95 (m, 21 H) ppm; ^{13}C NMR (101 MHz,

$CDCl_3$) δ 169.5 (C), 169.3(C), 152.1 (C), 140.8 (C), 137.7 (C), 131.5 (CH), 129.3 (2 X CH), 128.4 (CH), 128.1 (2 X CH), 128.1 (2 X CH), 127.9 (CH), 127.8 (CH), 126.9 (CH), 127.6 (2 X CH), 123.5 (C), 106.8 (CH), 89.4 (C), 76.9 (C), 62.2 (C), 61.4 (CH_2), 61.4 (CH_2), 46.7 (CH), 26.2 (CH_2), 18.0 (6 X CH_3), 14.0 (CH_3), 14.0 (CH_3), 12.7 (3 X CH) ppm. **Minor Isomer :** ^{1}H NMR (400 MHz, $CDCl_3$) δ 7.48 - 7.38 (m, 1 H), 7.35 (s, 1 H), 7.34 - 7.16 (m, 8 H), 7.13 (t, *J* = 3.2 Hz, 1 H), 6.88 (q, *J* = 3.0 Hz, 2 H), 5.78 (d, *J* = 10.4 Hz, 1 H), 4.87 (d, *J* = 10.5 Hz, 1 H), 4.28 - 4.04 (m, 4 H), 3.10 (d, *J* = 17.0 Hz, 1 H), 3.02 (d, *J* = 17.0 Hz, 1 H), 1.12 (t, *J* = 7.2 Hz, 6 H), 1.10 - 0.95 (m, 21 H) ppm; ^{13}C NMR (101 MHz, $CDCl_3$) δ 169.5 (C), 169.3 (C), 151.6 (C), 140.5 (C), 139.9 (C), 131.4 (CH), 129.7 (2 X CH), 128.4 (CH), 128.1 (2 X CH), 127.8 (CH), 127.8 (CH), 126.6 (CH), 126.6 (2 X CH), 126.1 (2 X CH), 123.8 (C), 108.7 (CH), 90.1 (C), 76.3 (C), 62.5 (C), 61.4 (CH_2), 61.4 (CH_2), 44.8 (CH), 25.5 (CH_2), 18.0 (6 X CH_3), 14.0 (CH_3), 14.0 (CH_3), 12.7 (3 X CH) ppm; IR (neat, cm^{-1}): 2942, 2866 1734; HRMS (TOF MS EI+) *m*/*z* calcd for $C_{38}H_{48}O_5SSi$ $[M]^+$: 644.2992, found: 644.3011.

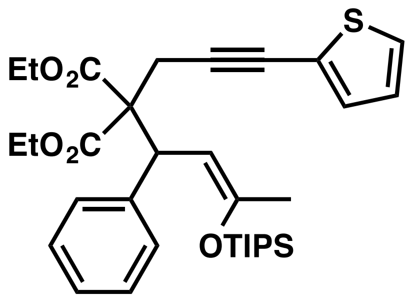

(11i): ^{1}H NMR (400 MHz, $CDCl_3$) δ 7.24 - 7.20 (m, 4 H), 7.19 - 7.13 (m, 2 H), 7.13 - 7.07 (m, 1 H), 6.92 (dd, *J* = 3.6, 5.1 Hz, 1 H), 5.36 (d, *J* = 10.7 Hz, 1 H), 4.30 (d, *J* = 10.8 Hz, 1 H), 4.27 - 4.01 (m, 4 H), 3.02 (d, *J* = 17.1 Hz, 1 H), 2.85 (d, *J* = 17.1 Hz, 1 H), 1.82 (s, 3 H), 1.25 (t, *J* = 7.2 Hz, 3 H), 1.18 (t, *J* = 7.1 Hz, 3 H), 1.08 - 1.00 (m, 21 H) ppm; ^{13}C NMR (101 MHz, $CDCl_3$) δ 169.6 (C), 169.4 (C), 150.6 (C), 140.6 (C), 131.3 (CH), 129.1 (2 X CH), 128.0 (2 X CH), 126.8 (CH), 126.8 (CH), 126.3 (CH), 123.7 (C), 105.3 (CH), 89.7 (C), 76.9 (C), 61.8(C), 61.4 (2 X CH_2), 47.0 (CH), 26.3 (CH_2), 18.2 (CH_3), 18.0 (6 X CH_3), 14.0

(CH_3), 14.0 (CH_3), 12.6 (3 X CH) ppm; IR (neat, cm^{-1}): 2943, 2866, 1725; HRMS (EI) *m*/*z* calcd for $C_{33}H_{46}O_5SSi$ $[M]^+$: 582.2835, found: 582.3091.

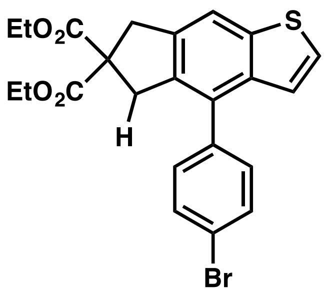

(12a): ^{1}H NMR (400 MHz, $CDCl_3$): δ 7.69 (d, *J* = 0.5 Hz, 1 H), 7.61 (td, *J* = 2.4, 8.6 Hz, 2 H), 7.32 (td, *J* = 2.3, 8.5 Hz, 2 H), 7.31 (s, 1 H), 7.07 (dd, *J* = 0.7, 5.5 Hz, 1 H), 4.18 (ddd, *J* = 0.7, 7.2, 14.2 Hz, 4 H), 3.73 (s, 2 H), 3.50 (s, 2 H), 1.23 (t, *J* = 7.1 Hz, 6 H) ppm; ^{13}C NMR (100 MHz, $CDCl_3$): δ 171.3 (2 X C), 139.6 (C), 137.9 (C), 137.8 (C), 137.5 (C), 135.2 (C), 131.9 (2 X CH), 131.7 (2 X CH), 131.0 (CH), 125.8 (CH), 122.7 (CH), 121.5 (C), 117.3 (CH), 61.8 (2 X CH_2), 61.0 (C), 40.3 (CH_2), 39.2 (CH_2), 14.0 (2 X CH_3) ppm; IR (neat, cm^{-1}): 1733; HRMS (EI) *m*/*z* calcd for $C_{23}H_{21}O_4SBr$ $[M]^+$: 472.0344, found: 472.0361.

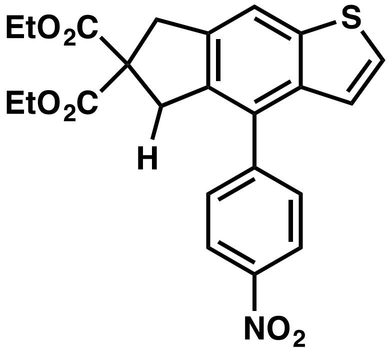

(12b): ^{1}H NMR (400 MHz, $CDCl_3$): δ 8.37 (td, *J* = 2.2, 9.0 Hz, 2 H), 7.76 (s, 1 H), 7.64 (td, *J* = 2.2, 9.0 Hz, 2 H), 7.39 (d, *J* = 5.5 Hz, 1 H), 7.05 (dd, *J* = 0.8, 5.6 Hz, 1 H), 4.19 (q, *J* = 7.1 Hz, 4 H), 3.76 (s, 2 H), 3.50 (s, 2 H), 1.24 (t, *J* = 7.1 Hz, 6 H) ppm; ^{13}C NMR (100 MHz, $CDCl_3$): δ 171.2 (2 X C), 147.2 (C), 145.9 (C), 139.8 (C), 137.6 (C), 137.4 (C), 135.3 (C), 130.8 (C), 130.3 (2 X CH), 126.6 (CH), 123.8 (2 X CH), 122.1 (CH), 118.2 (CH), 61.9 (2 X CH_2), 61.0 (C), 40.2 (CH_2), 39.2 (CH_2), 14.0 (2 X CH_3) ppm; IR (neat, cm^{-1}): 1733; HRMS (EI) *m*/*z* calcd for $C_{23}H_{21}NO_6S$ $[M]^+$: 439.1090, found: 439.1104.

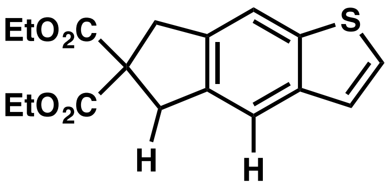

(12c): ^{1}H NMR (400 MHz, CDCl$_3$): δ 7.68 (s, 1 H), 7.62 (s, 1 H), 7.35 (d, *J* = 5.4 Hz, 1 H), 7.24 (dd, *J* = 0.7, 5.5 Hz, 1 H), 4.21 (q, *J* = 7.1 Hz, 4 H), 3.67 (d, *J* = 3.2 Hz, 4 H), 1.26 (t, *J* = 7.1 Hz, 6 H) ppm; ^{13}C NMR (100 MHz, CDCl$_3$): δ 171.6 (2 X C), 139.1 (C), 139.0 (C), 137.4 (C), 137.2 (C), 125.8 (CH), 123.4 (CH), 118.8 (CH), 117.8 (CH), 61.8 (2 X CH$_2$), 61.2 (C), 40.0(CH$_2$), 39.9 (CH$_2$), 14.2 (2 X CH$_3$) ppm; IR (neat, cm^{-1}): 1734; HRMS (EI) *m*/*z* calcd for $C_{17}H_{18}O_4S$ [M]$^+$: 318.0926, found: 318.0926.

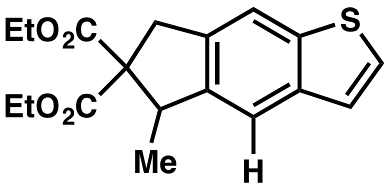

(12d): ^{1}H NMR (400 MHz, CDCl$_3$): δ 7.64 (s, 1 H), 7.58 (s, 1 H), 7.33 (d, *J* = 5.4 Hz, 1 H), 7.24 (d, *J* = 5.5 Hz, 1 H), 4.27 - 4.12 (m, 4 H), 4.08 (q, *J* = 7.2 Hz, 1 H), 3.84 (d, *J* = 16.7 Hz, 1 H), 3.38 (d, *J* = 16.6 Hz, 1 H), 1.30 (d, *J* = 7.2 Hz, 3 H), 1.24 (dt, *J* = 1.4, 7.1 Hz, 6 H) ppm; ^{13}C NMR (100 MHz, CDCl$_3$): δ 171.6 (C), 170.2 (C), 143.0 (C), 139.1 (C), 138.9 (C), 136.5 (C), 125.6 (CH), 123.6 (CH), 118.0 (CH), 117.6 (CH), 65.3 (C), 61.5 (CH$_2$), 61.3 (CH$_2$), 44.3 (CH), 38.6 (CH$_2$), 16.9 (CH$_3$), 14.2 (CH$_3$), 14.1 (CH$_3$) ppm; IR (neat, cm^{-1}): 2977, 1725; HRMS (EI) *m*/*z* calcd for $C_{18}H_{20}O_4S$ [M]$^+$: 332.1082, found: 332.1085.

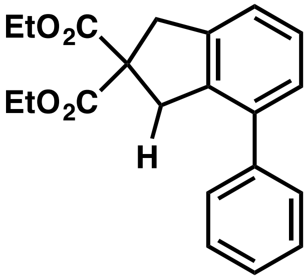

(12e): ^{1}H NMR (400 MHz, CDCl$_3$): δ 7.46 - 7.38 (m, 4 H), 7.37 - 7.29 (m, 1 H), 7.27 - 7.21 (m, 0 H), 7.20 - 7.13 (m, 2 H), 4.16 (ttd, *J* = 3.5, 7.0, 10.6 Hz, 4 H), 3.63 (d, *J* = 4.8 Hz, 4 H), 1.21 (t, *J* = 7.1 Hz, 6 H) ppm; ^{13}C NMR (100 MHz, CDCl$_3$): δ 171.6 (2 X C), 140.7 (C), 140.7 (C), 138.4 (C), 137.7 (C), 128.5 (2 X CH), 128.4 (2 X CH), 127.5 (2 X CH), 127.1 (CH), 123.2 (CH), 61.7 (2 X CH$_2$), 60.3 (C), 40.6 (CH$_2$), 40.2 (CH$_2$), 14.0

(2 X CH_3) ppm; IR (neat, cm^{-1}): 2984, 1734; HRMS (EI) *m/z* calcd for $C_{21}H_{22}O_4$ $[M]^+$: 338.1518, found: 338.1519.

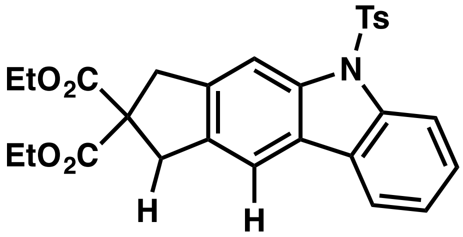

(12f): ^1H NMR (400 MHz, $CDCl_3$): δ 8.25 (d, *J* = 8.3 Hz, 1 H), 8.12 (s, 1 H), 7.80 (d, *J* = 7.3 Hz, 1 H), 7.65 (d, *J* = 8.5 Hz, 3 H), 7.41 (dt, *J* = 1.2, 7.9 Hz, 1 H), 7.33 - 7.26 (m, 1 H), 7.08 (d, *J* = 8.0 Hz, 2 H), 4.20 (q, *J* = 7.2 Hz, 4 H), 3.73 (s, 2 H), 3.65 (s, 2 H), 2.24 (s, 3 H), 1.34 - 1.17 (m, 6 H) ppm; ^{13}C NMR (100 MHz, $CDCl_3$): δ 171.5 (2 X C), 144.8 (C), 140.2 (C), 138.6 (C), 138.1 (C), 136.1 (2 X C), 135.0 (C), 129.7 (2 X CH), 127.0 (CH), 126.5 (2 X CH), 126.0 (C), 123.8 (CH), 119.7 (CH), 115.3 (CH), 115.2 (CH), 110.9 (CH), 61.8 (2 X CH_2), 61.0 (C), 40.8(CH_2), 40.0 (CH_2), 31.0, 21.5 (CH_3), 14.1(2 X CH_3) ppm; IR (neat, cm^{-1}): 1733; HRMS (EI) *m/z* calcd for $C_{28}H_{27}NO_6S$ $[M]^+$: 505.1559, found: 505.1561.

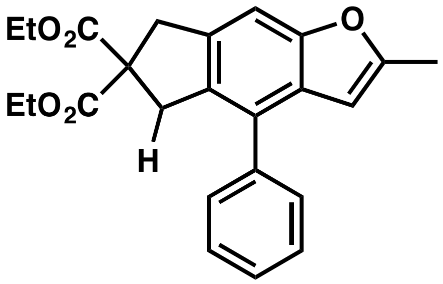

(12g): ^1H NMR (400 MHz, $CDCl_3$): δ 7.49 - 7.39 (m, 4 H), 7.39 - 7.30 (m, 1 H), 7.20 (d, *J* = 0.5 Hz, 1 H), 6.25 (t, *J* = 1.0 Hz, 1 H), 4.23 - 4.09 (m, 4 H), 3.68 (s, 2 H), 3.57 (s, 2 H), 2.38 (d, *J* = 0.9 Hz, 3 H), 1.21 (t, *J* = 7.2 Hz, 6 H) ppm; ^{13}C NMR (100 MHz, $CDCl_3$): δ 171.6 (2 X C), 155.4 (C), 154.7 (C), 138.7 (C), 136.0 (C), 132.0 (C), 129.7 (C_{quat}), 129.1 (2X CH), 128.4 (2 X CH), 127.7 (C), 127.1 (CH), 105.7 (CH), 102.1 (CH), 61.7(2 X CH_2), 61.1(C), 40.5(CH_2), 39.3 (CH_2), 17.7 (CH_3), 14.0 (2 X CH_3) ppm; IR (neat, cm^{-1}): 1736; HRMS (EI) *m/z* calcd for $C_{24}H_{24}O_5$ $[M]^+$: 392.1624, found: 392.1642.

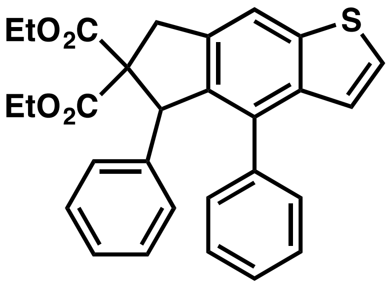

(12h): ^{1}H NMR (400 MHz, $CDCl_3$): δ 7.77 (s, 1 H), 7.54 (d, *J* = 7.5 Hz, 1 H), 7.43 (t, *J* = 7.4 Hz, 1 H), 7.27 - 7.21 (m, 1 H), 7.08 - 6.88 (m, 5 H), 6.65 (dd, *J* = 1.5, 7.9 Hz, 2 H), 6.53 (d, *J* = 7.5 Hz, 1 H), 5.21 (s, 1 H), 4.26 (d, *J* = 17.1 Hz, 1 H), 4.25 (qd, *J* = 7.2, 10.8 Hz, 1 H), 4.12 (qd, *J* = 7.1, 10.8 Hz, 1 H), 3.80 (qd, *J* = 7.2, 10.8 Hz, 1 H), 3.63 (qd, *J* = 7.2, 10.8 Hz, 1 H), 3.53 (d, *J* = 17.2 Hz, 1 H), 1.21 (t, *J* = 7.1 Hz, 3 H), 0.91 (t, *J* = 7.2 Hz, 3 H) ppm; ^{13}C NMR (100 MHz, $CDCl_3$): δ 171.8 (C), 168.8 (C), 140.0 (C), 139.7 (C), 139.6 (C), 138.9 (C), 138.4 (C), 136.8 (C), 134.4 (C), 129.2 (CH), 129.1 (CH), 128.9 (2 X CH), 128.2 (CH), 127.7 (2 X CH), 127.1 (CH), 126.7 (CH), 125.3 (CH), 123.3 (CH), 117.1 (CH), 66.5 (C), 61.8 (CH_2), 61.3 (CH_2), 54.9 (CH), 39.0 (CH_2), 14.0 (CH_3), 13.7 (CH_3) ppm; IR (neat, cm^{-1}): 2933, 2863, 1732; HRMS (EI) *m*/*z* calcd for $C_{29}H_{26}O_4S$ $[M]^+$: 470.1552, found: 470.1542.

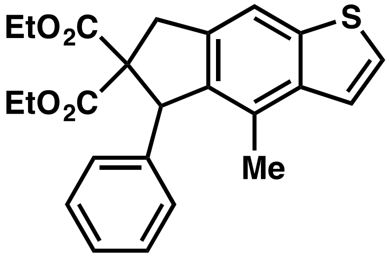

(12i): ^{1}H NMR (400 MHz, $CDCl_3$): δ 7.61 (s, 1 H), 7.30 (dd, *J* = 5.6, 19.2 Hz, 2 H), 7.21 - 7.09 (m, 3 H), 7.01 - 6.95 (m, 2 H), 5.34 (s, 1 H), 4.21 (dq, *J* = 7.2, 10.8 Hz, 1 H), 4.17 (d, *J* = 17.0 Hz, 1 H), 4.10 (dq, *J* = 7.2, 10.8 Hz, 1 H), 3.85 (qd, *J* = 7.1, 10.8 Hz, 1 H), 3.67 (qd, *J* = 7.2, 10.8 Hz, 1 H), 3.47 (d, *J* = 17.0 Hz, 1 H), 2.25 (s, 3 H), 1.21 (t, *J* = 7.1 Hz, 3 H), 1.01 (t, *J* = 7.2 Hz, 3 H) ppm; ^{13}C NMR (100 MHz, $CDCl_3$): δ 171.7 (C), 168.9 (C), 139.6 (C), 139.6 (C), 139.2 (C), 139.1 (C), 136.9 (C), 129.1 (2 X CH), 128.9 (C), 128.1 (2 X CH), 127.1 (CH), 125.1 (CH), 121.9 (CH), 115.3 (CH), 66.6 (C), 61.9 (CH_2), 61.3 (CH_2), 54.9 (CH), 38.9 (CH_2), 15.9 (CH_3), 14.0 (CH_3), 13.8 (CH_3) ppm; IR (neat, cm^{-1}): 2982, 1733; HRMS (EI) *m*/*z* calcd for $C_{24}H_{24}O_4S$ $[M]^+$: 408.1395, found: 408.1411.

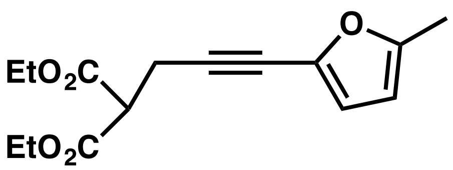

(15a): ^{1}H NMR (400 MHz, $CDCl_3$) δ 6.34 (d, *J* = 3.1 Hz, 1 H), 5.88 (dq, *J* = 1.1, 3.1 Hz, 1 H), 4.20 (q, *J* = 7.2 Hz, 4 H), 3.59 (t, *J* = 7.7 Hz, 1 H), 2.99 (d, *J* = 7.7 Hz, 2 H), 2.23 (s, 3 H), 1.25 (t, *J* = 7.2 Hz, 6 H) ppm; ^{13}C NMR (101 MHz , $CDCl_3$) δ 167.9 (2 X (C), 153.1 (C), 135.1 (C), 115.7 (CH), 106.7 (CH), 89.4 (C), 73.1 (C), 61.8 (2 X CH_2), 51.1 (CH), 19.5 (CH_2), 14.0 (2 X CH_3), 13.7 (CH_3) ppm; IR (neat, cm^{-1}) : 2988, 1733; HRMS (EI) *m/z* calcd for $C_{15}H_{18}O_5$ $[M]^+$: 278.1154, found: 278.1147.

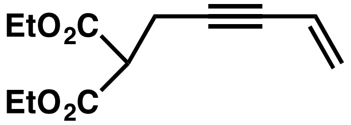

(15b): ^{1}H NMR (400 MHz, $CDCl_3$) δ 5.69 (ddt, *J* = 2.1, 11.1, 17.5 Hz, 1 H), 5.51 (dd, *J* = 2.1, 17.5 Hz, 1 H), 5.37 (dd, *J* = 2.2, 11.0 Hz, 1 H), 4.19 (q, *J* = 7.2 Hz, 4 H), 3.52 (t, *J* = 7.7 Hz, 1 H), 2.86 (dd, *J* = 1.9, 7.7 Hz, 2 H), 1.24 (t, *J* = 7.2 Hz, 6 H) ppm; ^{13}C NMR (101 MHz, $CDCl_3$) δ 168.0 (2 X C), 126.6 (CH_2), 117.0 (CH), 86.1 (C), 81.1 (C), 61.7 (2 X CH_2), 51.3 (CH), 19.3 (CH_2), 14.0 (2 X CH_3) ppm; IR (neat, cm^{-1}): 2984, 1732; HRMS (EI) *m/z* calcd for $C_{12}H_{14}O_4$ $[M]^+$: 224.1049, found: 224.1010.

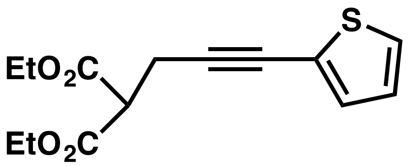

(15c): ^{1}H NMR (400 MHz, $CDCl_3$) δ 7.16 (dd, *J* = 1.1, 5.2 Hz, 1 H), 7.09 (dd, *J* = 0.9, 3.5 Hz, 1 H), 6.90 (dd, *J* = 3.6, 5.2 Hz, 1 H), 4.22 (q, *J* = 7.1 Hz, 4 H), 3.61 (t, *J* = 7.7 Hz, 1 H), 3.00 (d, *J* = 7.7 Hz, 2 H), 1.27 (t, *J* = 7.2 Hz, 6 H). ^{13}C NMR (101 MHz, $CDCl_3$) δ 168.0 (2 X C), 131.6 (CH), 126.8 (CH), 126.5 (CH), 123.3 (C), 89.5 (C), 75.6 (C), 61.8 (2 X CH_2), 51.2 (CH), 19.7 (CH_2), 14.1 (2 X CH_3) ppm; IR (neat, cm^{-1}): 2980, 1732; HRMS (EI) *m/z* calcd for $C_{14}H_{16}O_4S[M]^+$: 280.7069, found: 280.0753.

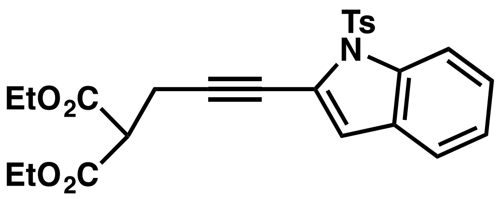

(15d): ^{1}H NMR (400 MHz, $CDCl_3$) $\delta = 8.16$ (dq, J = 0.7, 8.5 Hz, 1 H), 7.86 - 7.78 (m, 2 H), 7.40 (td, J = 0.9, 7.8 Hz, 1 H), 7.32 (ddd, J = 1.3, 7.2, 8.5 Hz, 1 H), 7.23 - 7.18 (m, 3 H), 6.76 (s, 1 H), 4.31 - 4.18 (m, 4 H), 3.73 (t, J = 7.6 Hz, 1 H), 3.13 (d, J = 7.6 Hz, 2 H), 2.33 (s, 3 H), 1.27 (t, J = 7.2 Hz, 6 H) ppm; ^{13}C NMR (101 MHz, $CDCl_3$) δ 168.0 (2 X C), 144.9 (C), 136.2 (C), 135.7 (C), 129.7 (2 X CH), 128.9 (C), 127.1 (2 X CH), 125.7 (CH), 123.8 (CH), 120.9 (CH), 120.7 (C), 117.0 (CH), 114.7 (CH), 93.7 (C), 73.4 (C), 62.0 (2 X CH_2), 51.0 (CH), 21.6 (CH_3), 19.9 (CH_2), 14.1 (2 X CH_3) ppm; IR (neat, cm^{-1}): 2986, 1733; HRMS (EI) m/z calcd for $C_{25}H_{25}O_6NS$ $[M]^+$: 467.1403, found: 467.1374.

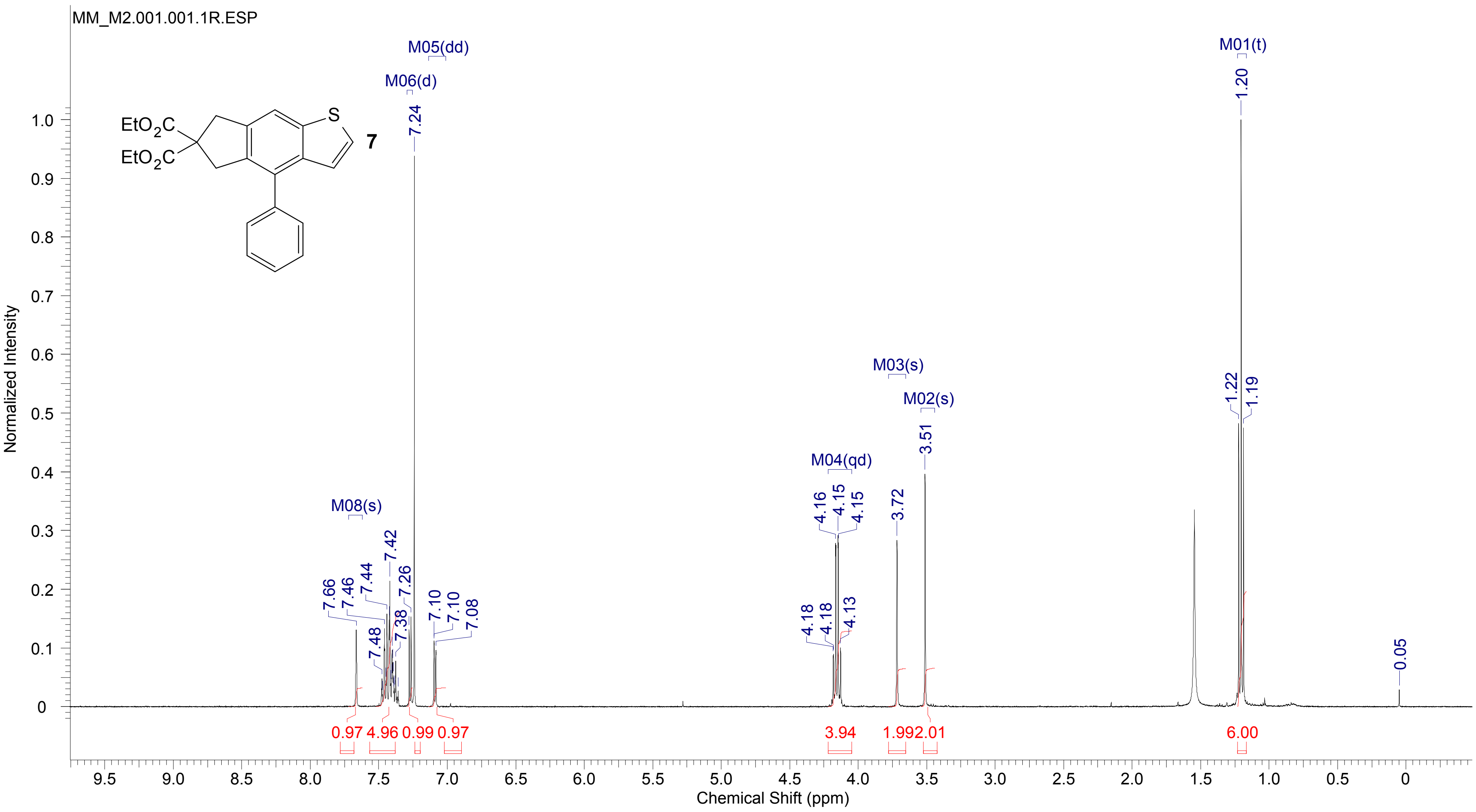
MM_M2.001.001.1R.ESP
EtO2C
EtO2C
S
7
M05(dd)
M06(d)
M01(t)
M03(s)
M02(s)
M04(qd)
M08(s)
Normalized Intensity
Chemical Shift (ppm)
0.97 4.96 0.99 0.97
3.94
1.99 2.01
6.00

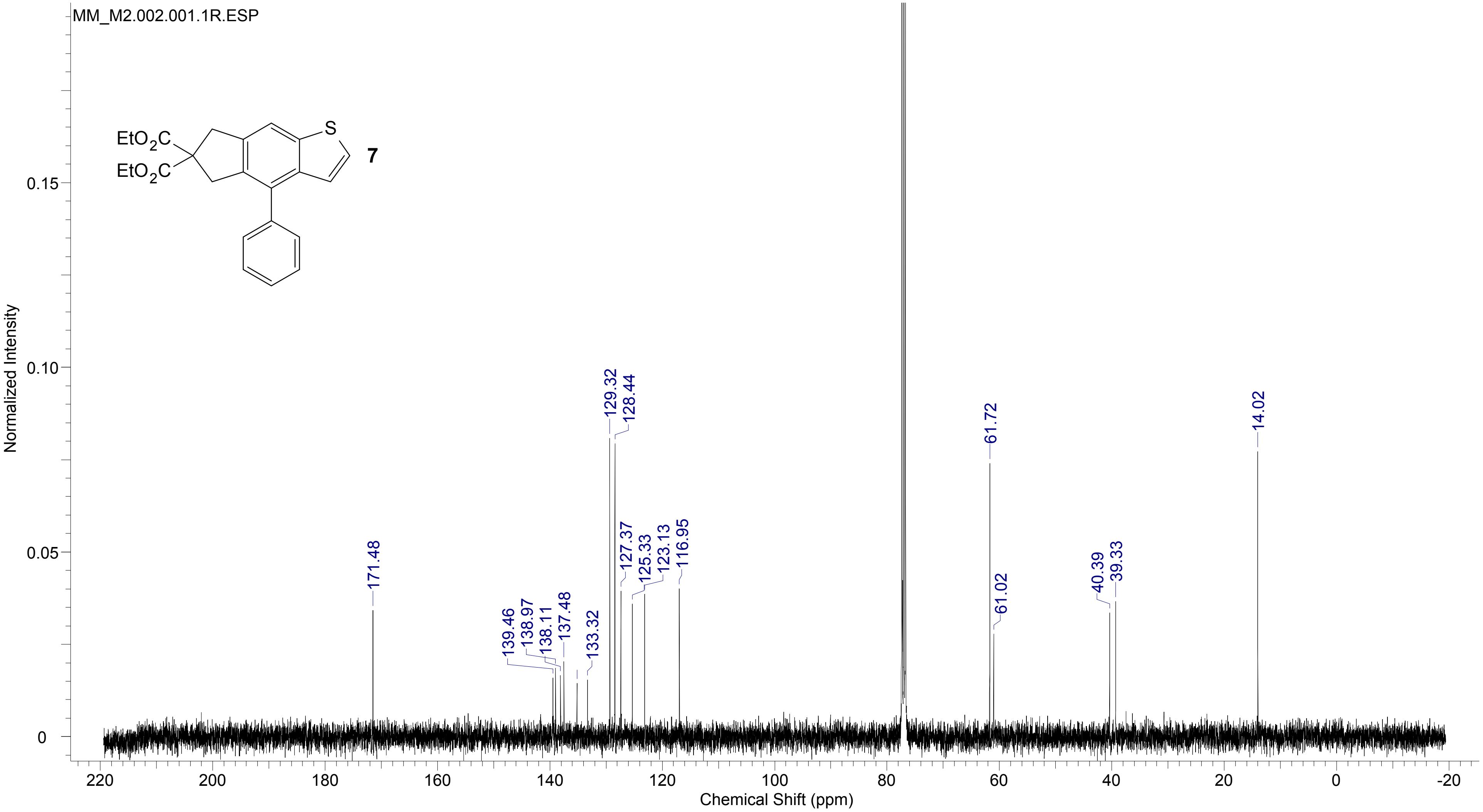
MM_M2.002.001.1R.ESP
EtO2C
EtO2C
S
7
171.48
139.46
138.97
138.11
137.48
133.32
129.32
128.44
127.37
125.33
123.13
116.95
61.72
61.02
40.39
39.33
14.02
Normalized Intensity
0.15
0.10
0.05
0
220
200
180
160
140
120
100
80
60
40
20
0
-20
Chemical Shift (ppm)

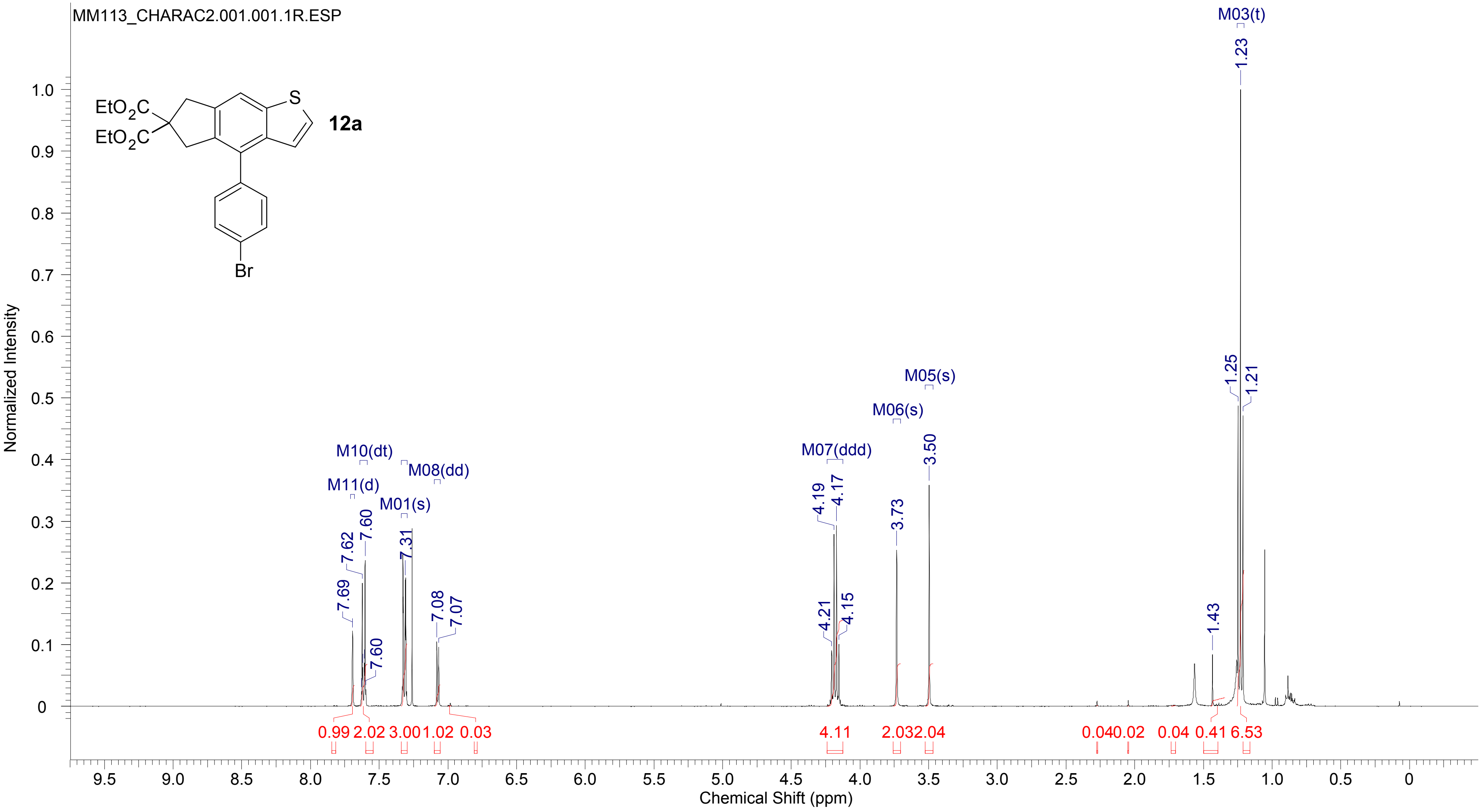
MM113_CHARAC2.001.001.1R.ESP
EtO2C
EtO2C
S
Br
12a
M03(t)
1.23
M05(s)
M06(s)
M07(ddd)
M10(dt)
M08(dd)
M11(d)
M01(s)
1.25
1.21
3.50
3.73
4.19
4.17
4.21
4.15
7.69
7.62
7.60
7.31
7.08
7.07
1.43
0.99
2.02
3.00
1.02
0.03
4.11
2.03
2.04
0.04
0.02
0.04
0.41
6.53
Normalized Intensity
Chemical Shift (ppm)

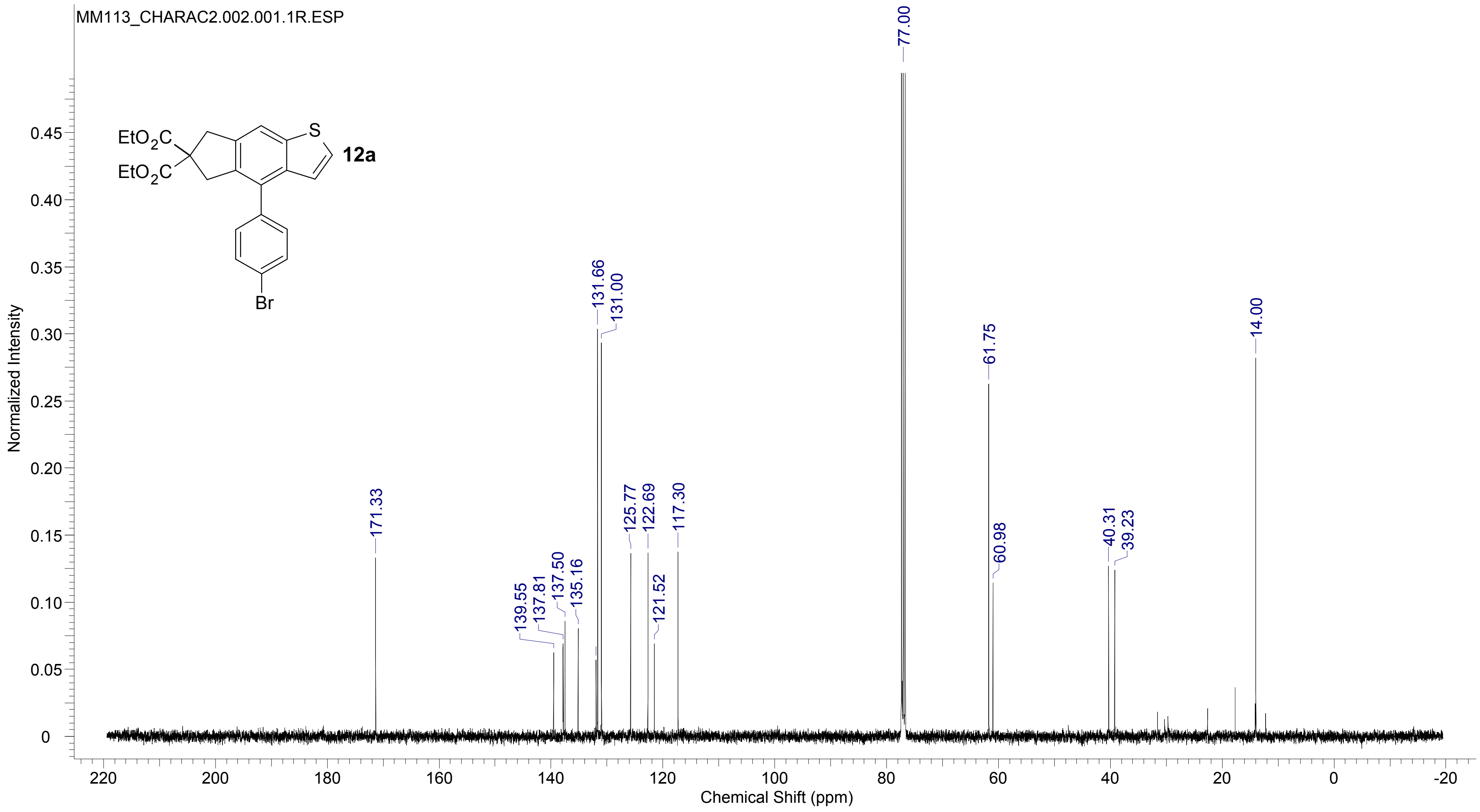
MM113_CHARAC2.002.001.1R.ESP
EtO2C
EtO2C
S
12a
Br
77.00
131.66
131.00
171.33
139.55
137.81
137.50
135.16
125.77
122.69
121.52
117.30
61.75
60.98
40.31
39.23
14.00
Normalized Intensity
Chemical Shift (ppm)

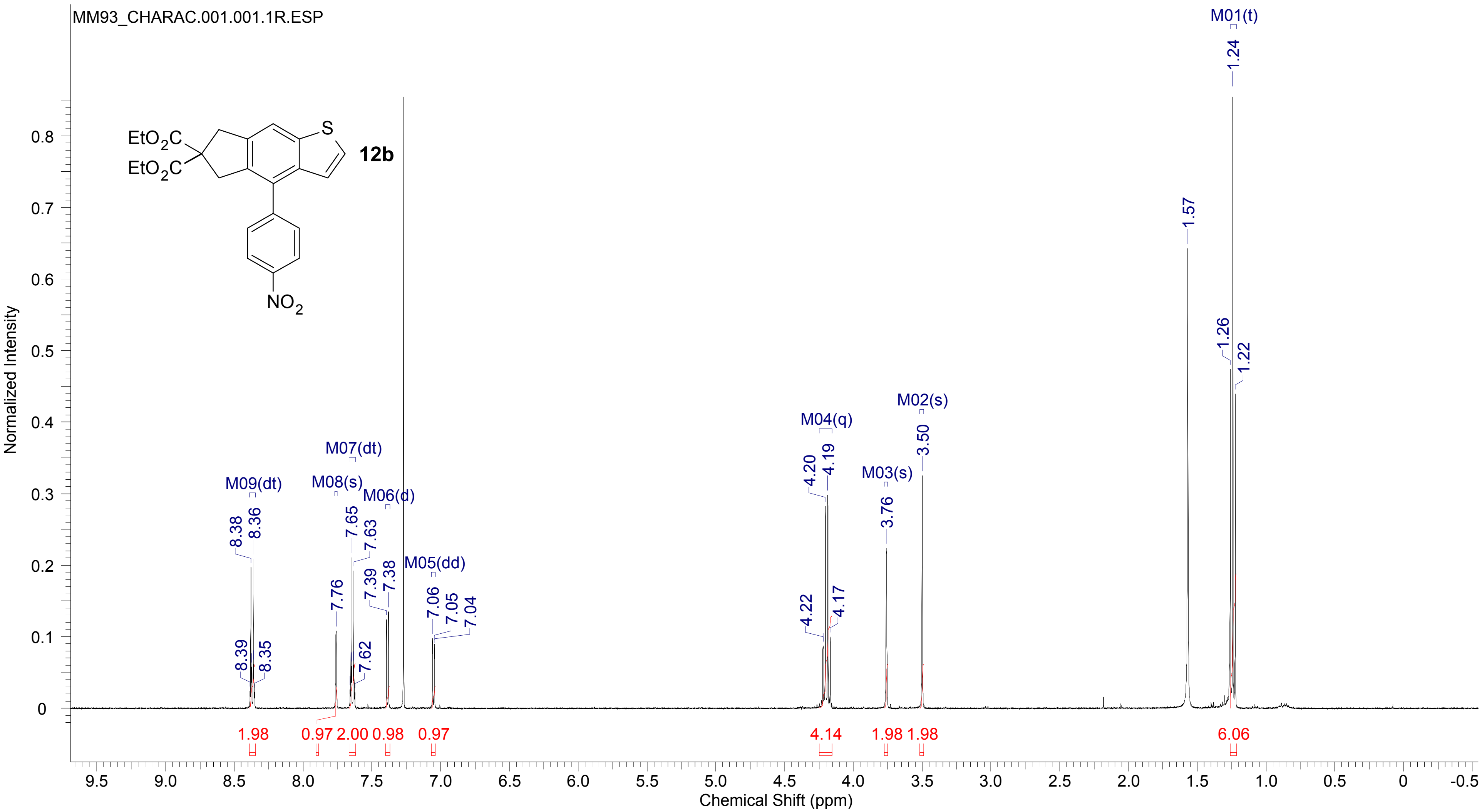
MM93_CHARAC.001.001.1R.ESP
EtO2C
EtO2C
S
NO2
12b
M01(t)
1.24
1.57
1.26
1.22
M02(s)
3.50
M03(s)
3.76
M04(q)
4.20
4.19
4.22
4.17
M05(dd)
7.06
7.05
7.04
M06(d)
7.39
7.38
M07(dt)
7.65
7.63
7.62
M08(s)
7.76
M09(dt)
8.38
8.36
8.39
8.35
1.98
0.97
2.00
0.98
0.97
4.14
1.98
1.98
6.06
Normalized Intensity
Chemical Shift (ppm)

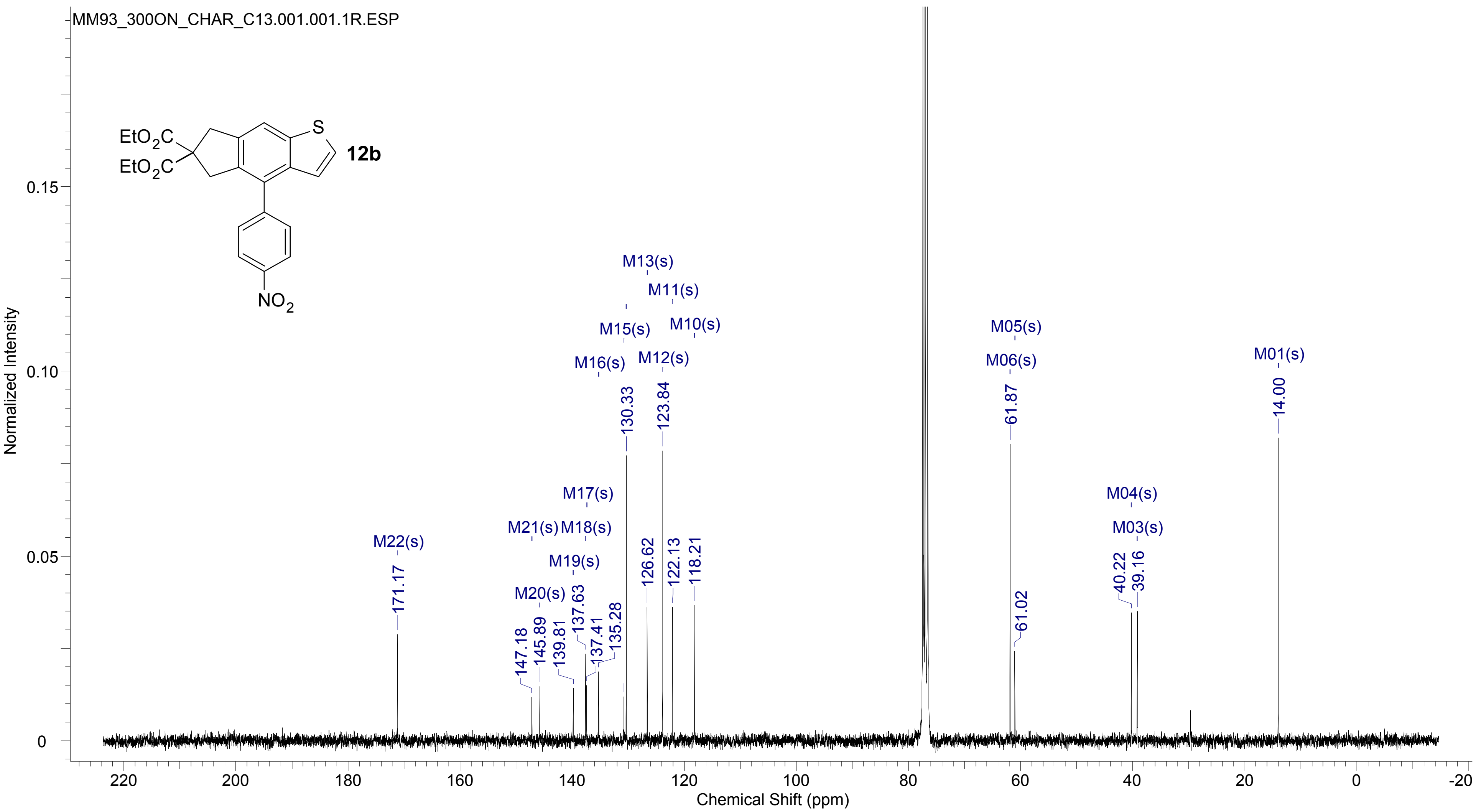
MM93_300ON_CHAR_C13.001.001.1R.ESP
EtO2C
EtO2C
S
NO2
12b
M22(s)
171.17
M21(s)
M20(s)
147.18
145.89
M19(s)
139.81
M18(s)
M17(s)
137.63
137.41
M16(s)
135.28
M15(s)
130.33
M13(s)
126.62
M12(s)
123.84
M11(s)
122.13
M10(s)
118.21
M05(s)
M06(s)
61.87
61.02
M04(s)
M03(s)
40.22
39.16
M01(s)
14.00
Normalized Intensity
0.15
0.10
0.05
0
220
200
180
160
140
120
100
80
60
40
20
0
-20
Chemical Shift (ppm)

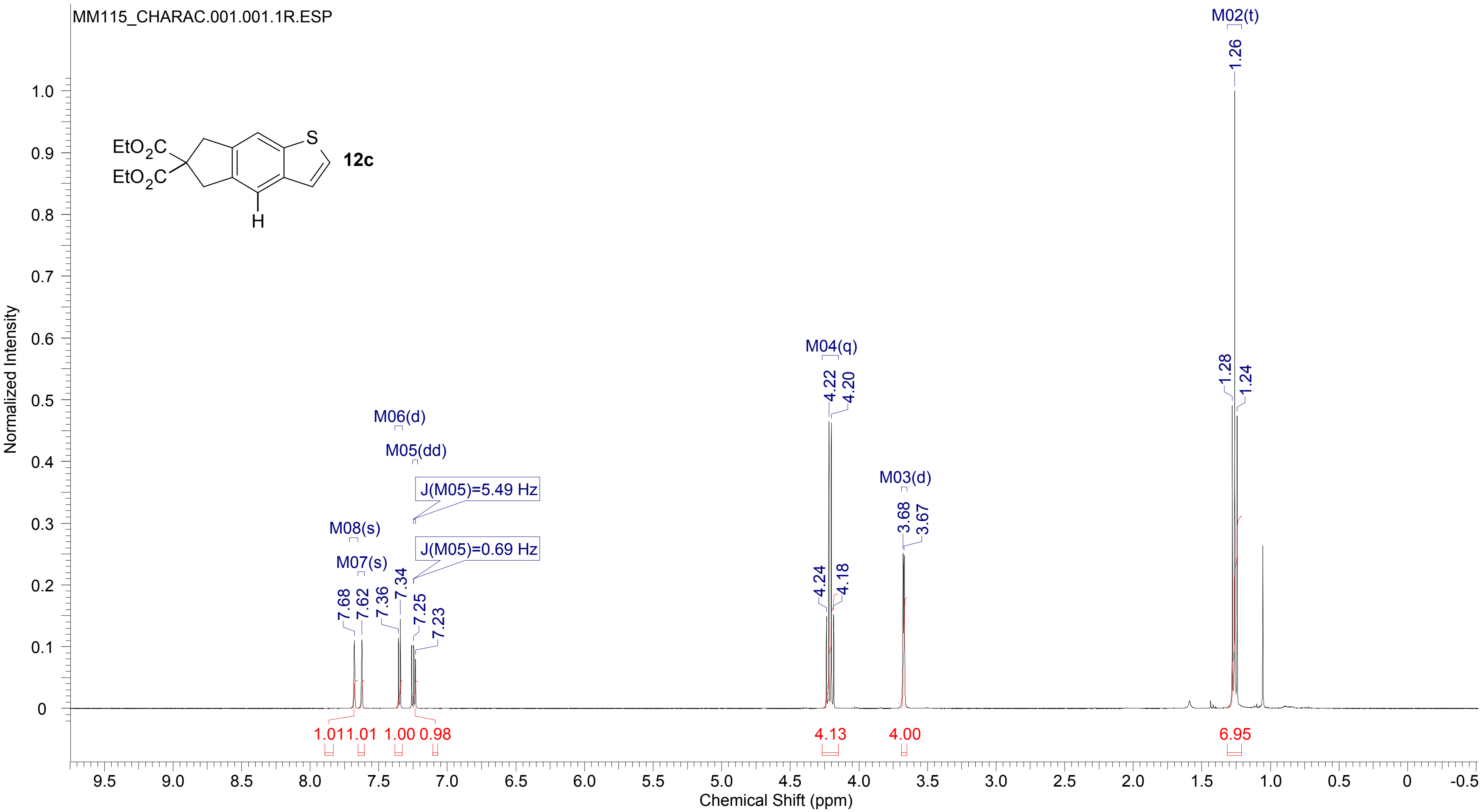

MM115_CHARAC.001.001.1R.ESP
EtO2C
EtO2C
S
H
12c
M02(t)
1.26
1.28
1.24
M04(q)
4.22
4.20
4.24
4.18
M03(d)
3.68
3.67
M06(d)
M05(dd)
J(M05)=5.49 Hz
J(M05)=0.69 Hz
M08(s)
M07(s)
7.68
7.62
7.36
7.34
7.25
7.23
1.01
1.01
1.00
0.98
4.13
4.00
6.95
Normalized Intensity
Chemical Shift (ppm)

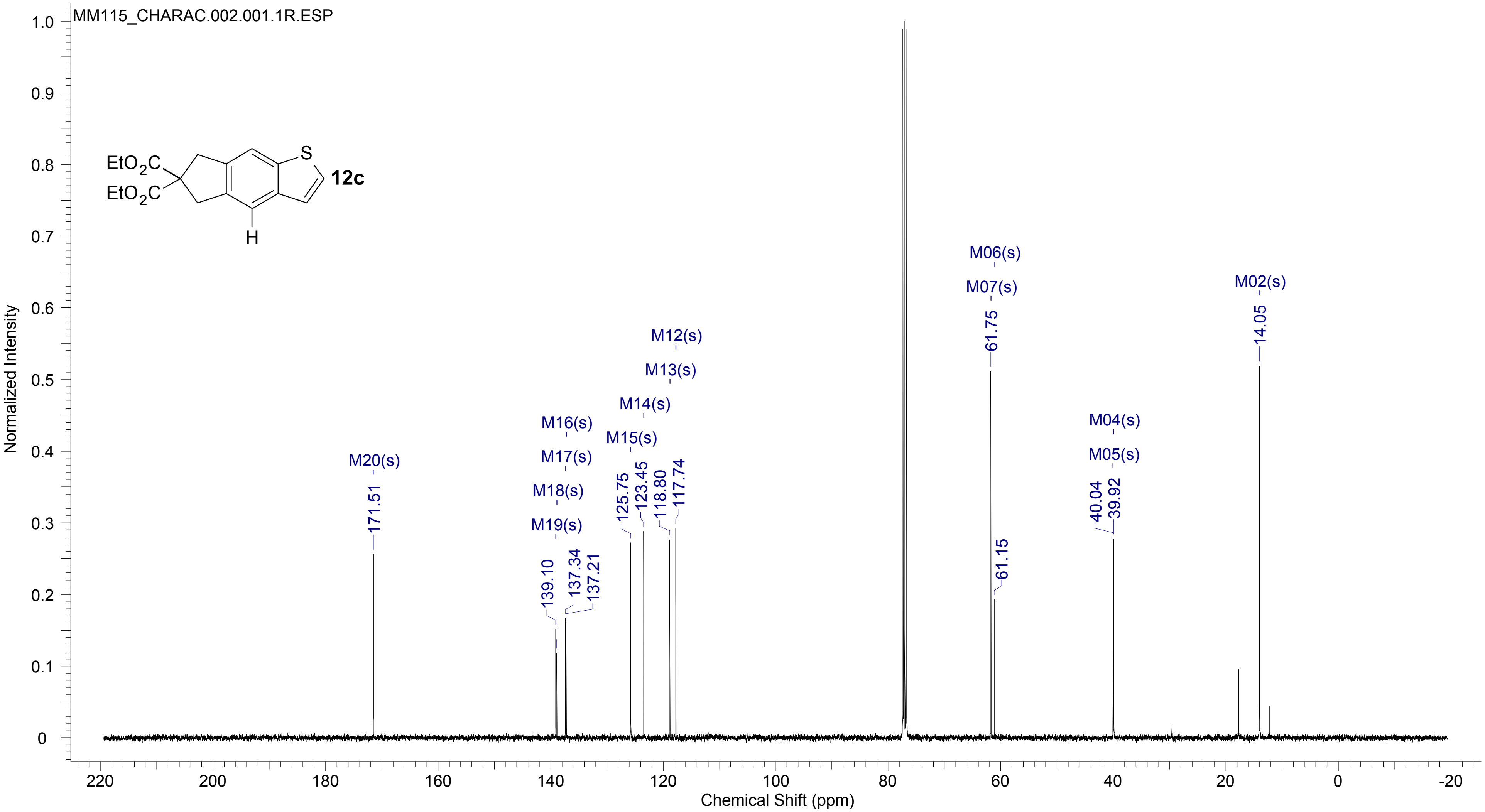
MM115_CHARAC.002.001.1R.ESP
EtO2C
EtO2C
S
H
12c
M20(s)
171.51
M16(s)
M17(s)
M18(s)
M19(s)
139.10
137.34
137.21
M15(s)
125.75
M14(s)
123.45
M13(s)
118.80
M12(s)
117.74
M06(s)
M07(s)
61.75
61.15
M04(s)
M05(s)
40.04
39.92
M02(s)
14.05
Normalized Intensity
Chemical Shift (ppm)

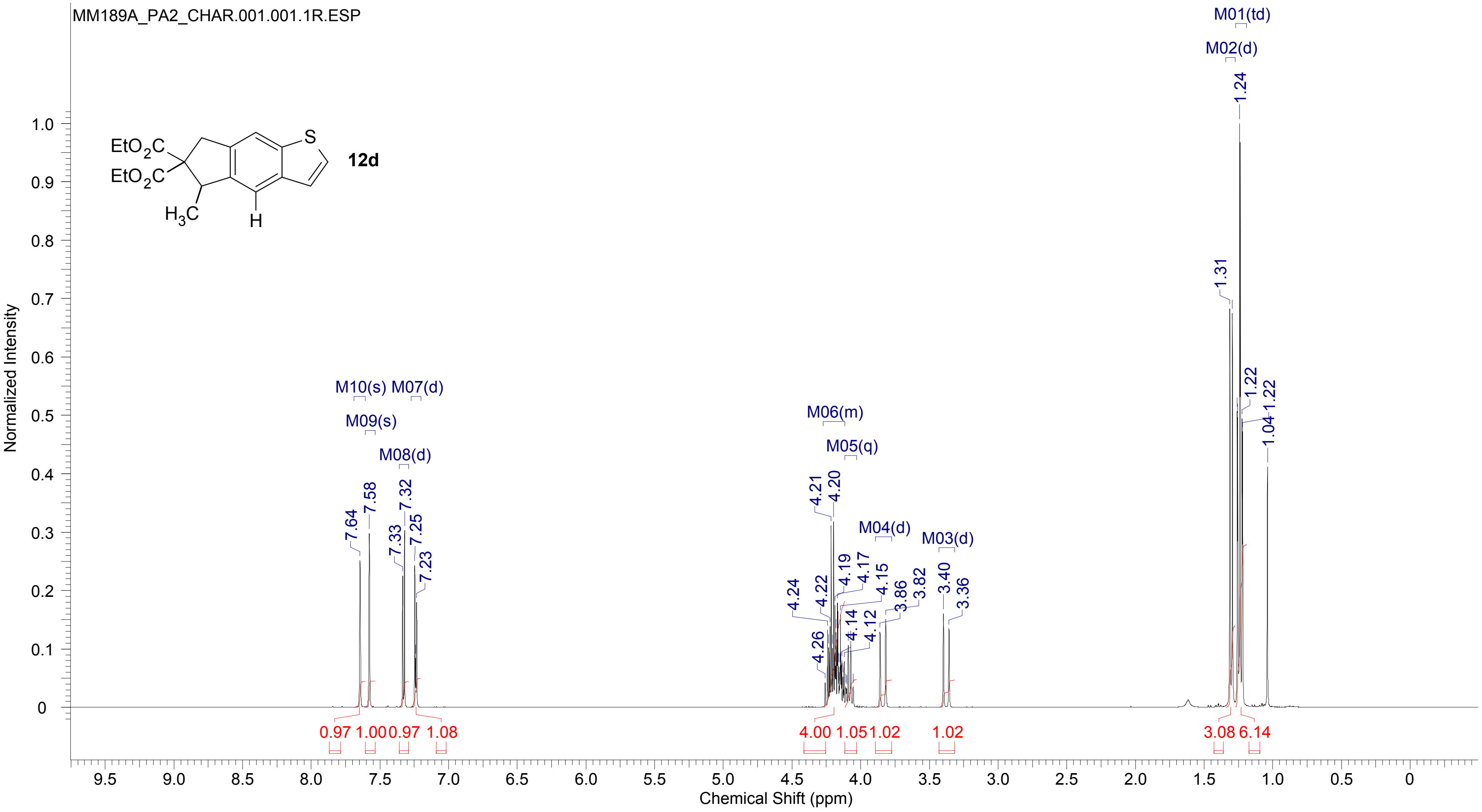
MM189A_PA2_CHAR.001.001.1R.ESP
EtO2C
EtO2C
S
H3C
H
12d
M01(td)
M02(d)
M03(d)
M04(d)
M05(q)
M06(m)
M07(d)
M08(d)
M09(s)
M10(s)
7.64
7.58
7.33
7.32
7.25
7.23
4.26
4.24
4.22
4.21
4.20
4.19
4.17
4.15
4.14
4.12
3.86
3.82
3.40
3.36
1.31
1.24
1.22
1.22
1.04
0.97
1.00
0.97
1.08
4.00
1.05
1.02
1.02
3.08
6.14
Normalized Intensity
Chemical Shift (ppm)

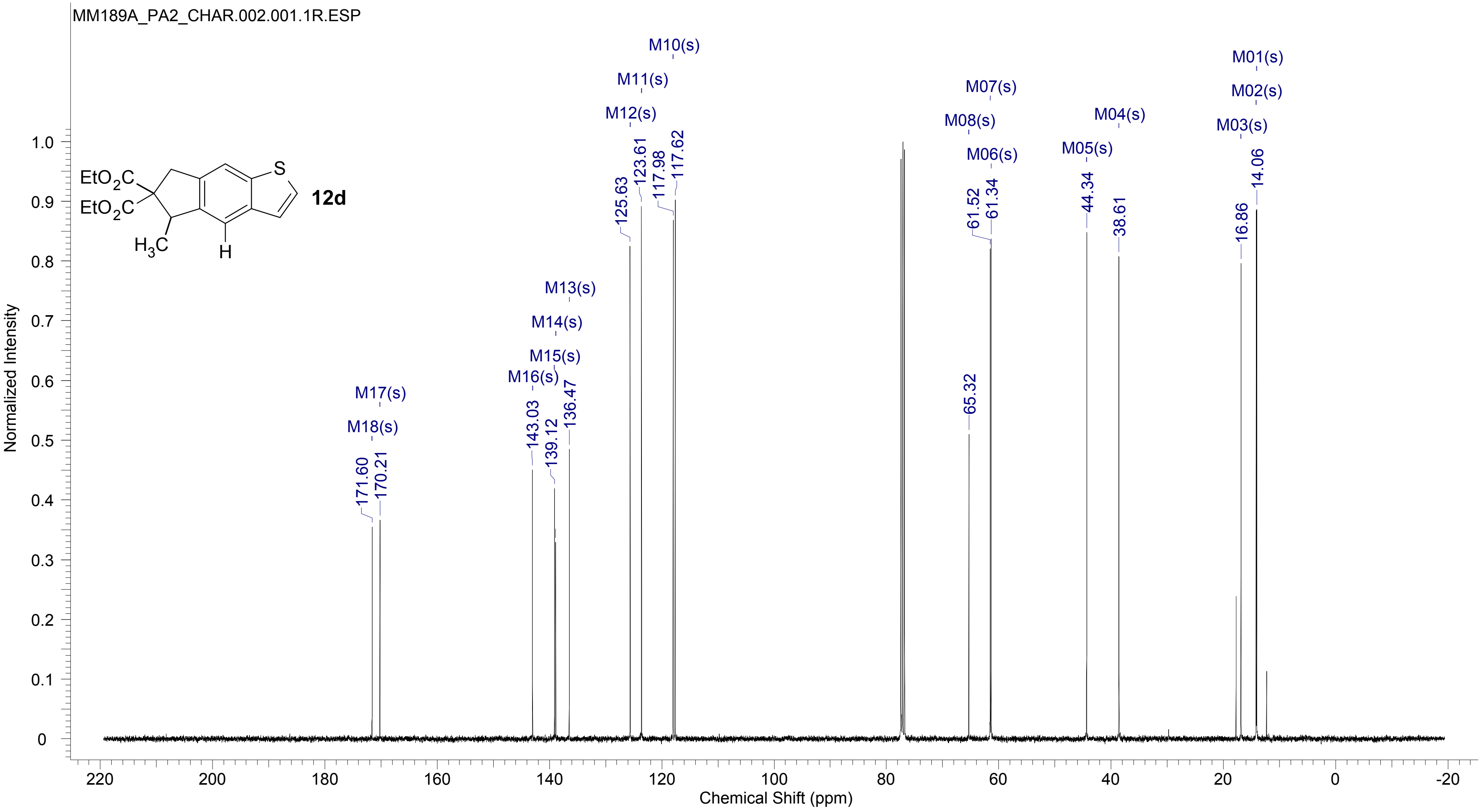

MM189A_PA2_CHAR.002.001.1R.ESP
12d
EtO2C
EtO2C
H3C
S
H
M01(s)
M02(s)
M03(s)
M04(s)
M05(s)
M06(s)
M07(s)
M08(s)
M10(s)
M11(s)
M12(s)
M13(s)
M14(s)
M15(s)
M16(s)
M17(s)
M18(s)
171.60
170.21
143.03
139.12
136.47
125.63
123.61
117.98
117.62
65.32
61.52
61.34
44.34
38.61
16.86
14.06
Normalized Intensity
Chemical Shift (ppm)

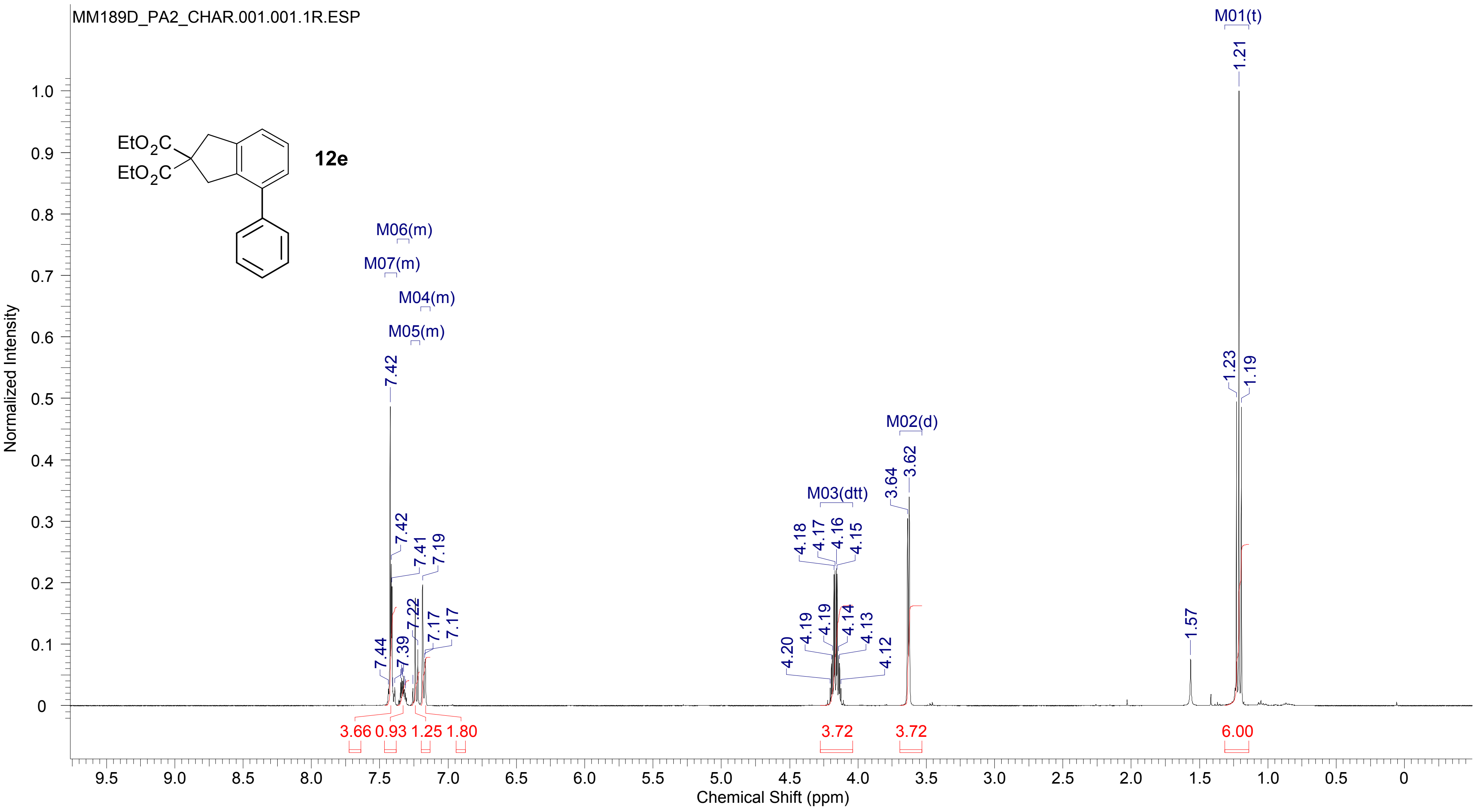

MM189D_PA2_CHAR.001.001.1R.ESP
EtO2C
EtO2C
12e
M01(t)
M02(d)
M03(dtt)
M04(m)
M05(m)
M06(m)
M07(m)
Normalized Intensity
Chemical Shift (ppm)
3.66 0.93 1.25 1.80
3.72
3.72
6.00

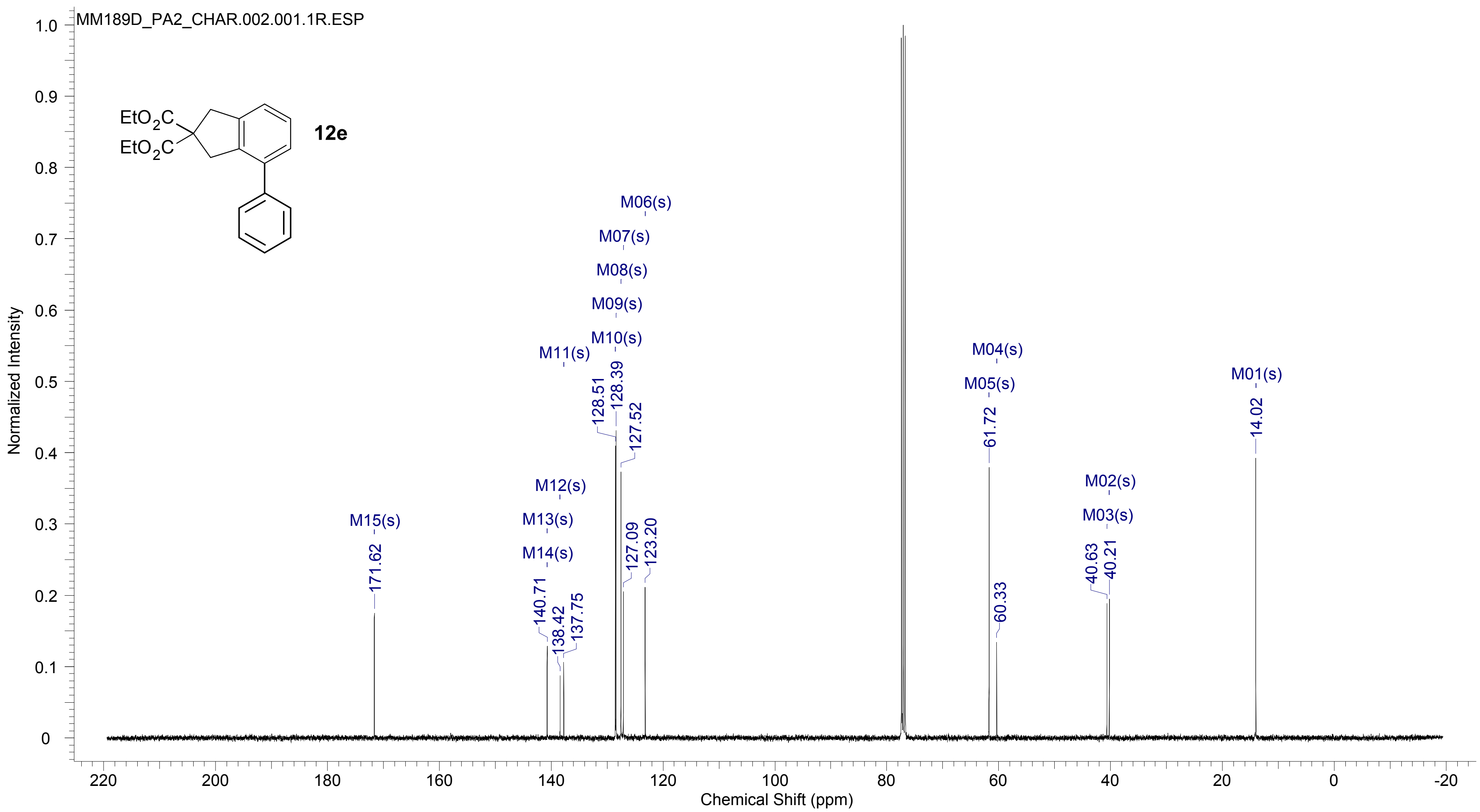
MM189D_PA2_CHAR.002.001.1R.ESP
EtO2C
EtO2C
12e
M15(s)
171.62
M11(s)
M12(s)
M13(s)
M14(s)
140.71
138.42
137.75
M06(s)
M07(s)
M08(s)
M09(s)
M10(s)
128.51
128.39
127.52
127.09
123.20
M04(s)
M05(s)
61.72
60.33
M02(s)
M03(s)
40.63
40.21
M01(s)
14.02
Normalized Intensity
Chemical Shift (ppm)

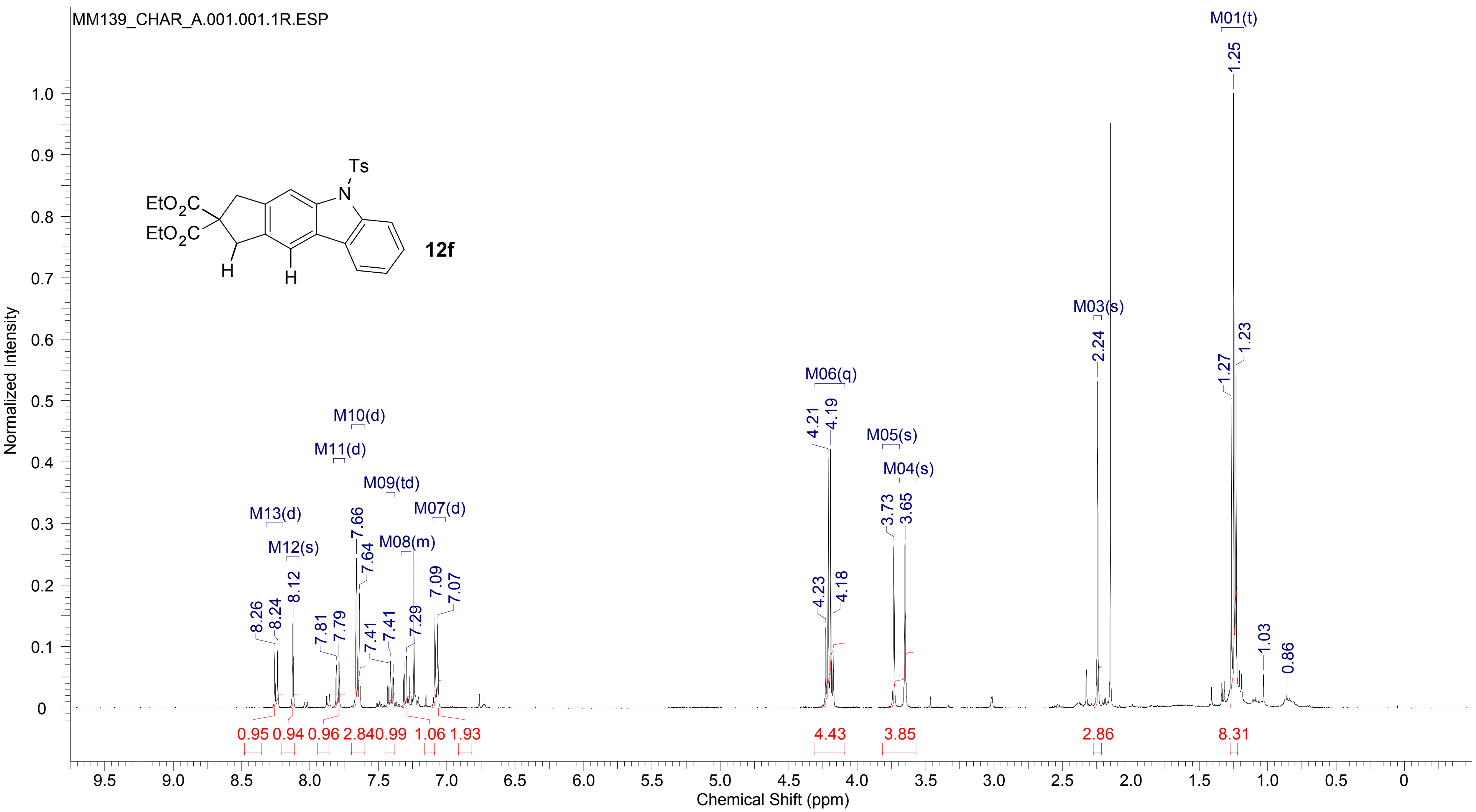
MM139_CHAR_A.001.001.1R.ESP
Ts
N
EtO2C
EtO2C
H
H
12f
M01(t)
M03(s)
M06(q)
M05(s)
M04(s)
M10(d)
M11(d)
M09(td)
M13(d)
M12(s)
M08(m)
M07(d)
0.95 0.94 0.96 2.84 0.99 1.06 1.93
4.43
3.85
2.86
8.31
Normalized Intensity
Chemical Shift (ppm)

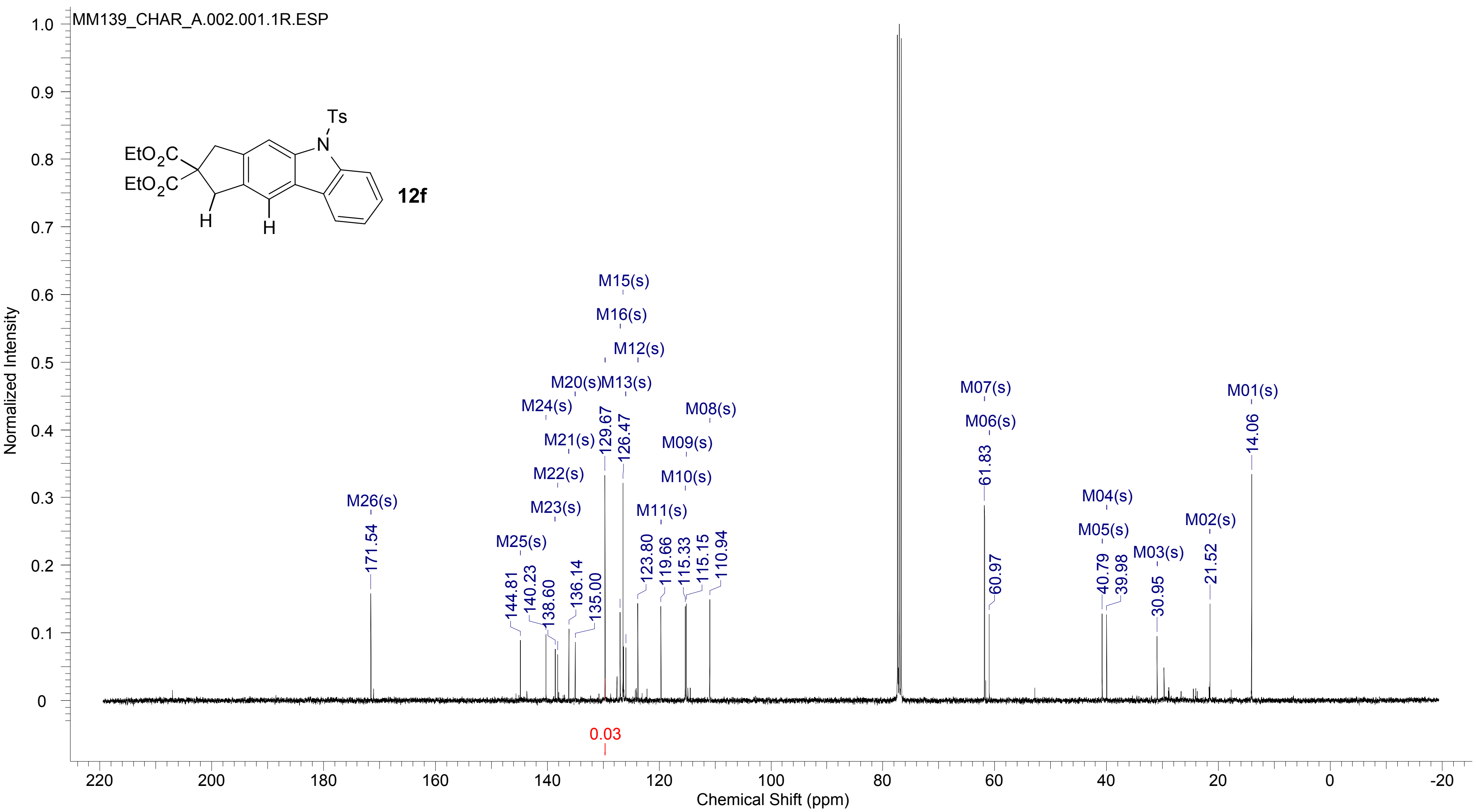
MM139_CHAR_A.002.001.1R.ESP
Ts
N
EtO2C
EtO2C
H
H
12f
Normalized Intensity
Chemical Shift (ppm)
M26(s)
171.54
M25(s)
144.81
140.23
138.60
136.14
135.00
129.67
126.47
123.80
119.66
115.33
115.15
110.94
61.83
60.97
40.79
39.98
30.95
21.52
14.06
0.03

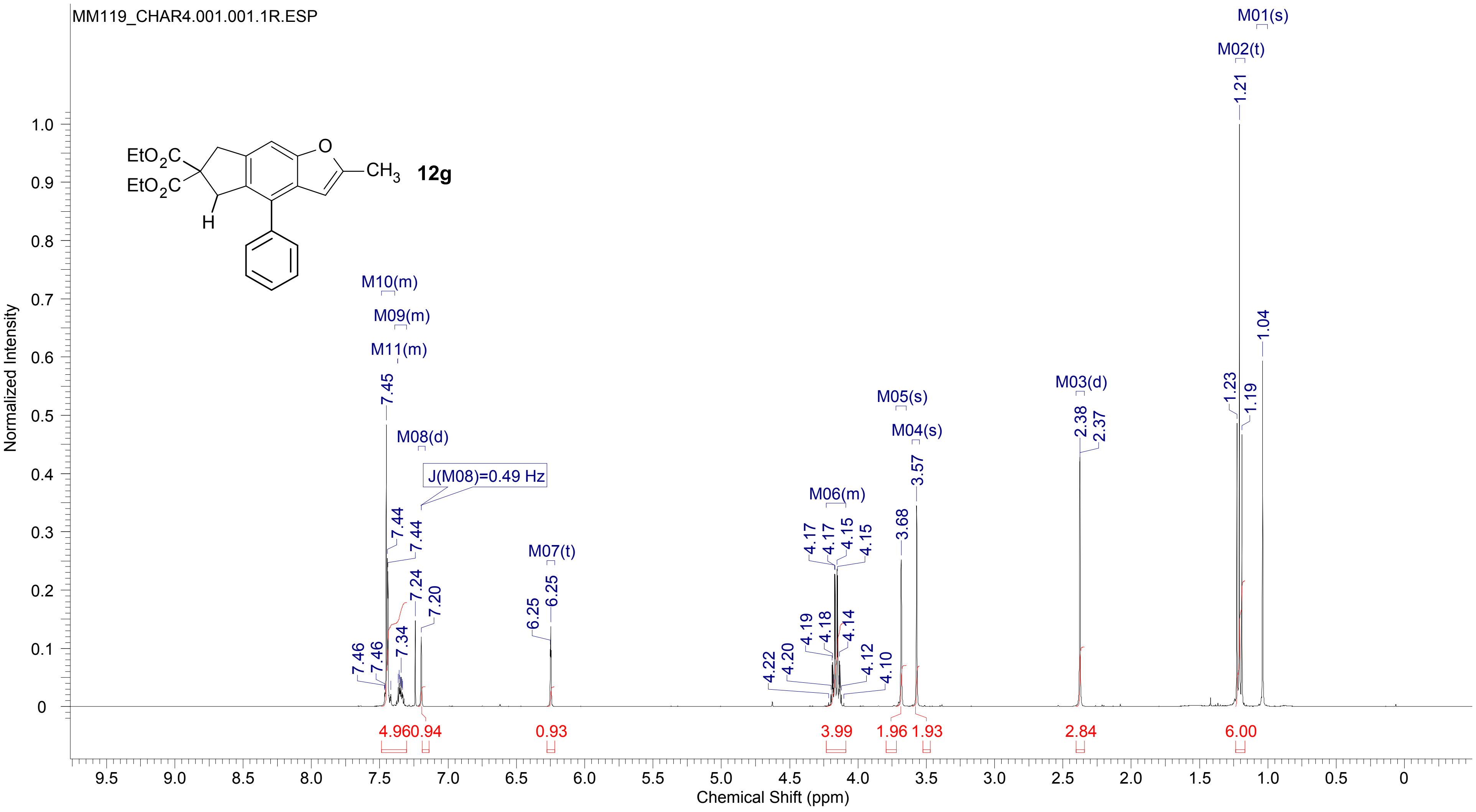
MM119_CHAR4.001.001.1R.ESP
12g
Normalized Intensity
Chemical Shift (ppm)
J(M08)=0.49 Hz

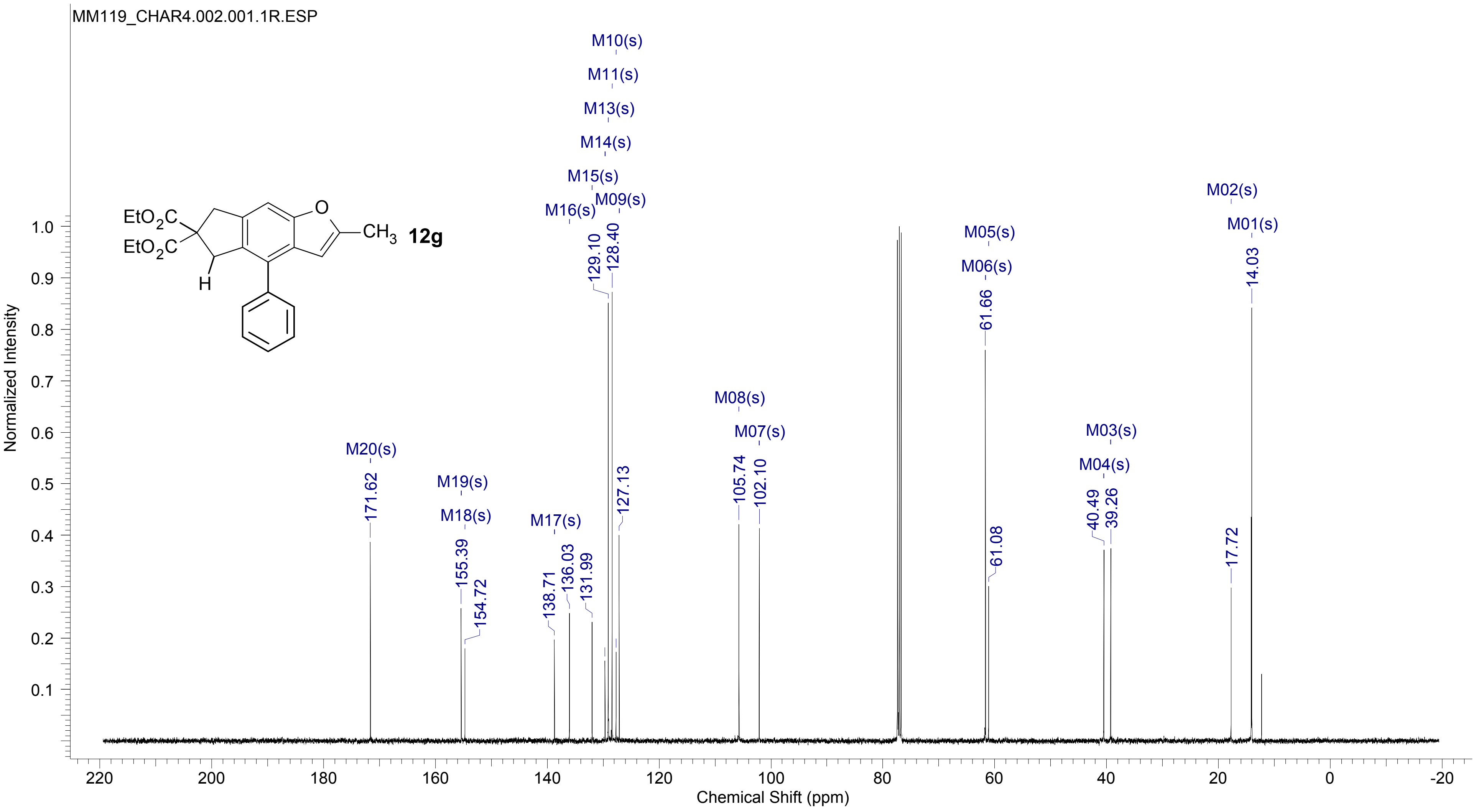
MM119_CHAR4.002.001.1R.ESP
EtO2C
EtO2C
H
O
CH3
12g
M10(s)
M11(s)
M13(s)
M14(s)
M15(s)
M16(s)
M09(s)
129.10
128.40
127.13
M02(s)
M01(s)
14.03
M05(s)
M06(s)
61.66
61.08
M08(s)
M07(s)
105.74
102.10
M03(s)
M04(s)
40.49
39.26
17.72
M20(s)
171.62
M19(s)
M18(s)
155.39
154.72
M17(s)
138.71
136.03
131.99
Normalized Intensity
Chemical Shift (ppm)

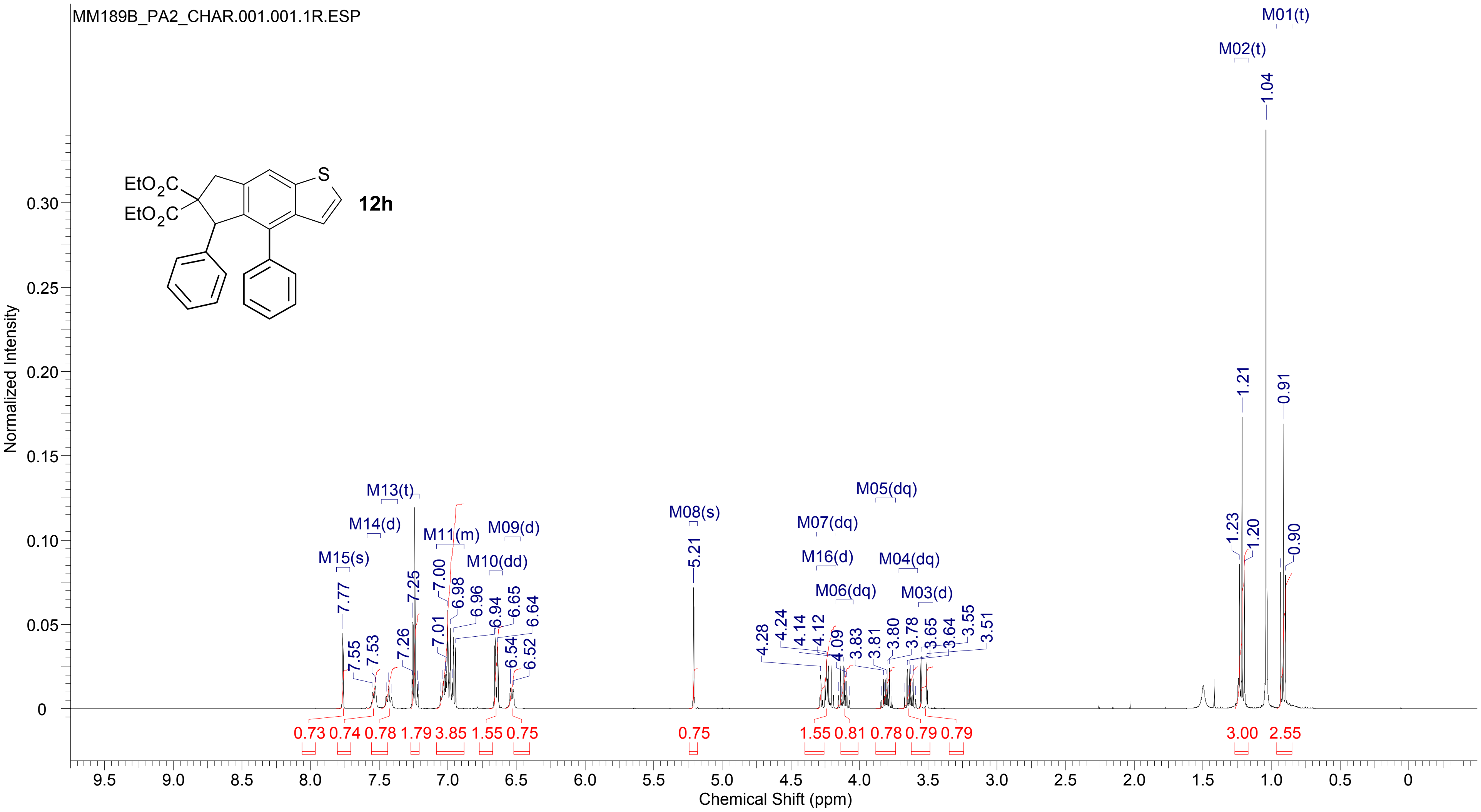

MM189B_PA2_CHAR.001.001.1R.ESP
12h
EtO2C
EtO2C
S
M01(t)
M02(t)
1.04
1.21
0.91
1.23
1.20
0.90
M15(s)
M14(d)
M13(t)
M11(m)
M09(d)
M10(dd)
M08(s)
M07(dq)
M16(d)
M06(dq)
M05(dq)
M04(dq)
M03(d)
7.77
7.55
7.53
7.26
7.25
7.01
7.00
6.98
6.96
6.94
6.65
6.64
6.54
6.52
5.21
4.28
4.24
4.14
4.12
4.09
3.83
3.81
3.80
3.78
3.65
3.64
3.55
3.51
0.73
0.74
0.78
1.79
3.85
1.55
0.75
0.75
1.55
0.81
0.78
0.79
0.79
3.00
2.55
Normalized Intensity
Chemical Shift (ppm)

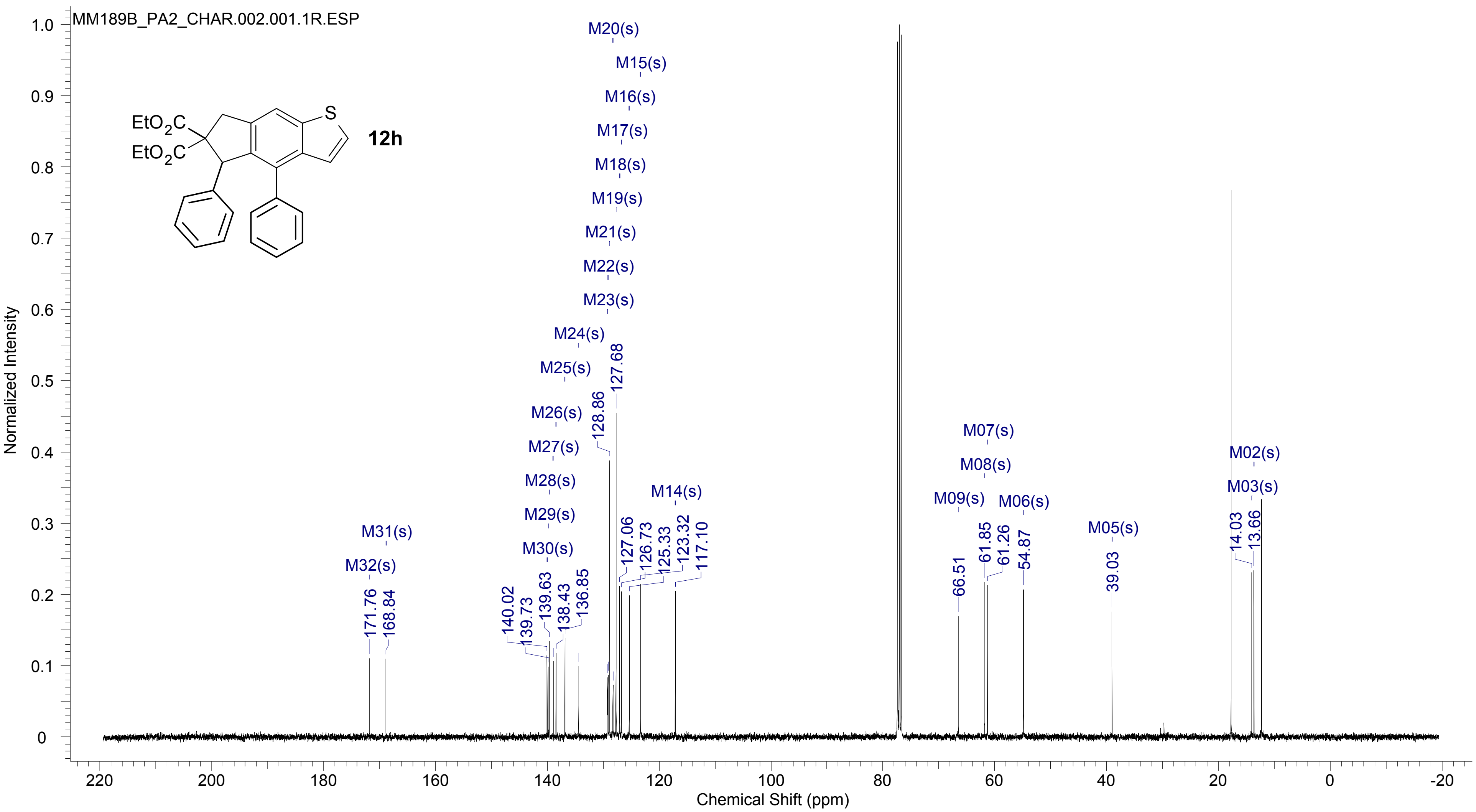
MM189B_PA2_CHAR.002.001.1R.ESP
12h
EtO2C
EtO2C
S
M20(s)
M15(s)
M16(s)
M17(s)
M18(s)
M19(s)
M21(s)
M22(s)
M23(s)
M24(s)
M25(s)
M26(s)
M27(s)
M28(s)
M29(s)
M30(s)
M31(s)
M32(s)
M14(s)
M07(s)
M08(s)
M09(s)
M06(s)
M05(s)
M02(s)
M03(s)
171.76
168.84
140.02
139.73
139.63
138.43
136.85
128.86
127.68
127.06
126.73
125.33
123.32
117.10
66.51
61.85
61.26
54.87
39.03
14.03
13.66
Normalized Intensity
Chemical Shift (ppm)

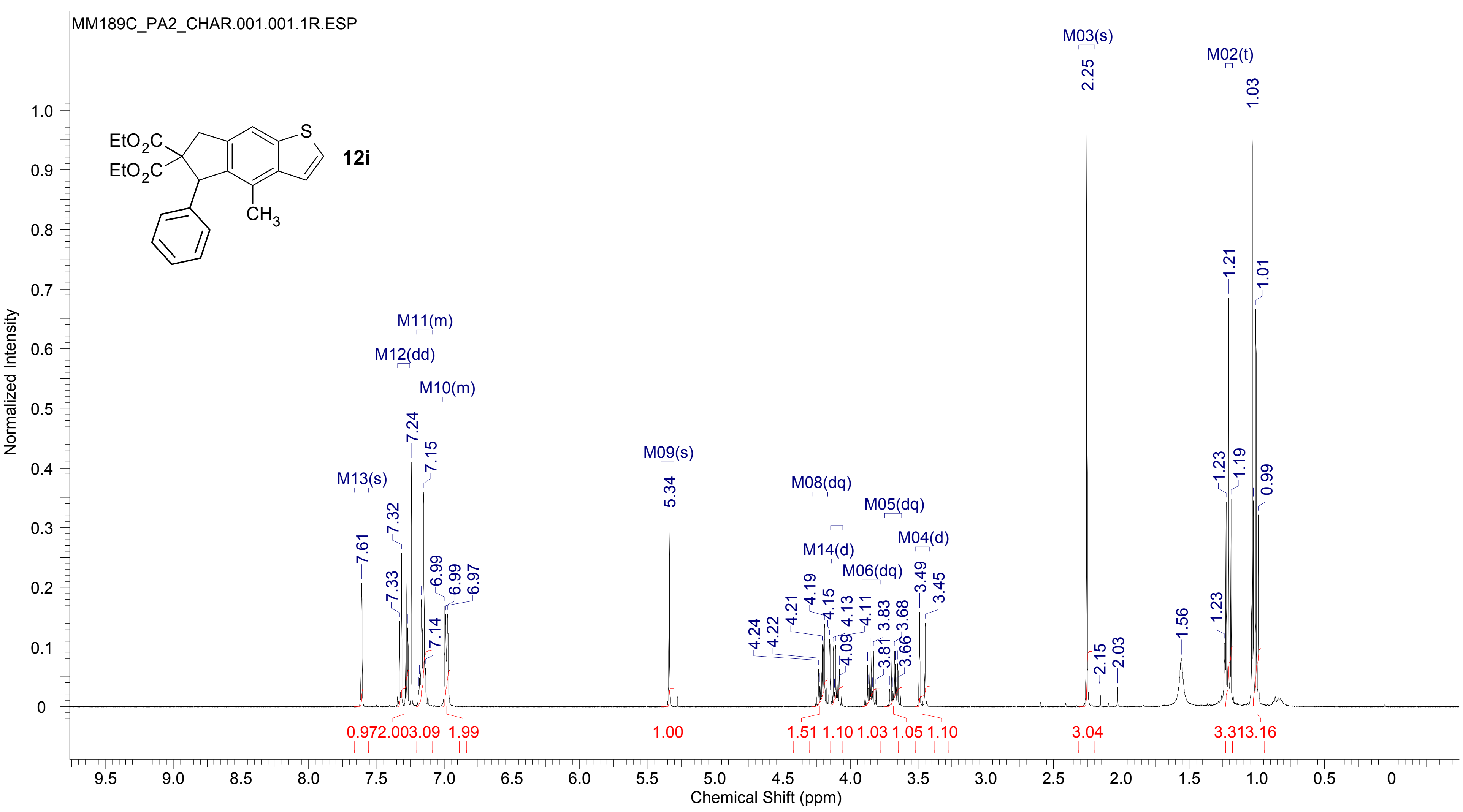
MM189C_PA2_CHAR.001.001.1R.ESP
EtO2C
EtO2C
S
CH3
12i
M03(s)
2.25
M02(t)
1.03
1.21
1.01
1.23
1.19
0.99
1.23
1.56
2.15
2.03
M13(s)
7.61
7.33
7.32
M12(dd)
M11(m)
7.24
M10(m)
7.15
7.14
6.99
6.99
6.97
M09(s)
5.34
M08(dq)
M14(d)
M06(dq)
M05(dq)
M04(d)
4.24
4.22
4.21
4.19
4.15
4.13
4.11
4.09
3.83
3.81
3.68
3.66
3.49
3.45
0.97
2.00
3.09
1.99
1.00
1.51
1.10
1.03
1.05
1.10
3.04
3.31
3.16
Normalized Intensity
Chemical Shift (ppm)

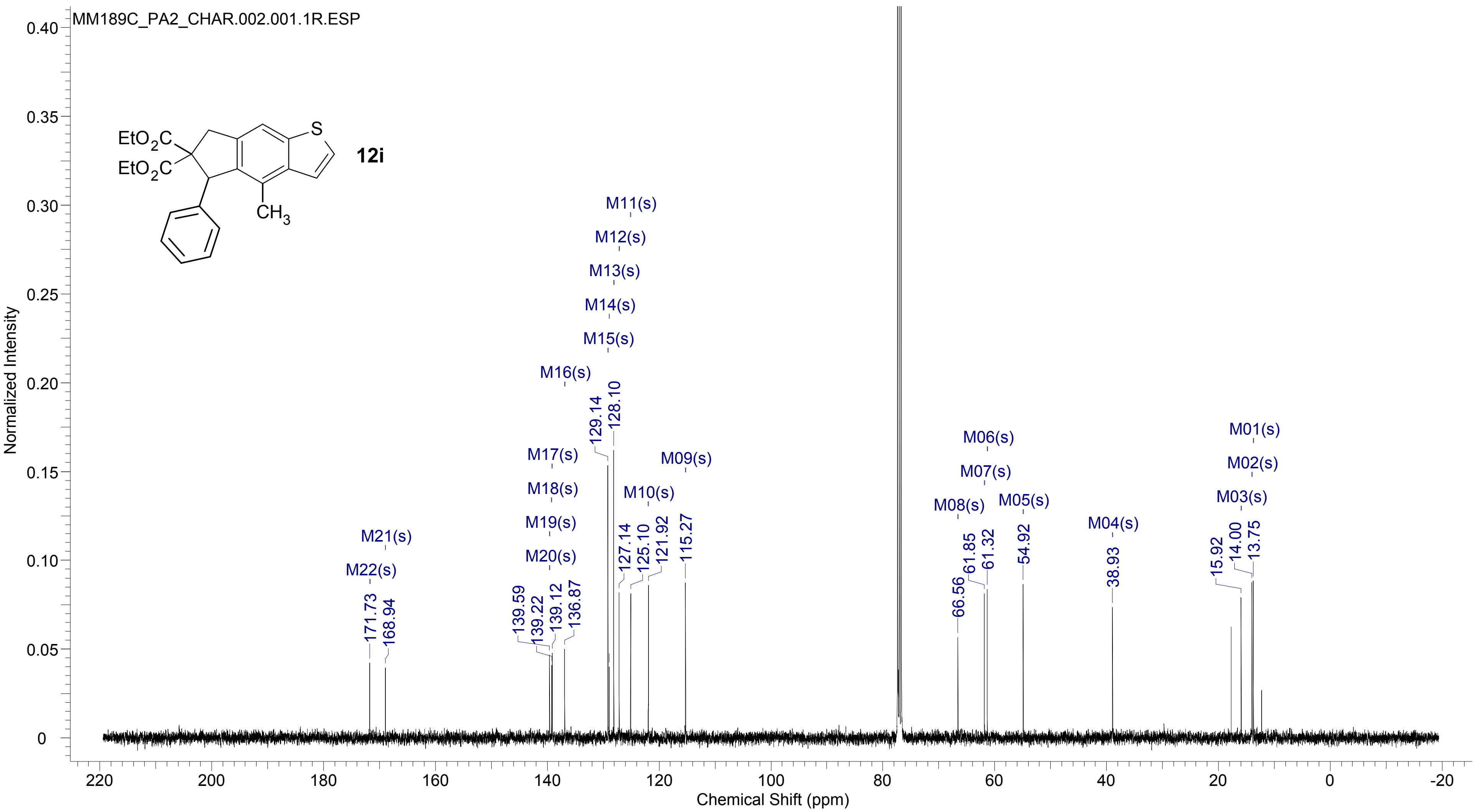
MM189C_PA2_CHAR.002.001.1R.ESP
EtO2C
EtO2C
S
CH3
12i
M01(s)
M02(s)
M03(s)
M04(s)
M05(s)
M06(s)
M07(s)
M08(s)
M09(s)
M10(s)
M11(s)
M12(s)
M13(s)
M14(s)
M15(s)
M16(s)
M17(s)
M18(s)
M19(s)
M20(s)
M21(s)
M22(s)
171.73
168.94
139.59
139.22
139.12
136.87
129.14
128.10
127.14
125.10
121.92
115.27
66.56
61.85
61.32
54.92
38.93
15.92
14.00
13.75
Normalized Intensity
Chemical Shift (ppm)
